# RCC2 and CD24 cooperate to modulate prostate cancer progression through vimentin ubiquitination and **β**-catenin activation

**DOI:** 10.1172/JCI192883

**Published:** 2025-10-15

**Authors:** Xuelian Cui, Yicun Wang, Chao Zhang, Zhichao Liu, Haiyan Yu, Lizhong Wang, Jiangbing Zhou, Runhua Liu

**Affiliations:** 1 Department of Genetics and; 2 O’Neal Comprehensive Cancer Center, University of Alabama at Birmingham, Birmingham, Alabama, USA.; 3 Department of Neurosurgery and; 4 Biomedical Engineering, Yale University, New Haven, Connecticut, USA.

**Keywords:** Cell biology, Genetics, Oncology, Prostate cancer

## Abstract

CD24 promotes prostate cancer progression and metastasis by disrupting the ARF-NPM interaction and impairing p53 signaling. However, the mechanisms underlying CD24-driven metastasis remain unclear. This study identifies a novel interaction between CD24 and Regulator of Chromosome Condensation 2 (RCC2), a protein involved in cell proliferation and migration. IHC analysis of prostate adenocarcinoma samples showed frequent coexpression of CD24 (49%) and RCC2 (82%) with a positive correlation between coexpression of CD24 (49%) and RCC2 (82%). Functional assays revealed complex roles: RCC2 KO suppressed proliferation but increased migration and invasion, while CD24 KO reduced both proliferation and migration. Dual KO of CD24 and RCC2 further inhibited proliferation but had varied effects on migration. In mouse xenografts, RCC2 KO increased lung metastasis without significantly affecting primary tumor growth, while CD24 KO reduced both tumor growth and metastasis. Mechanistically, RCC2 controls migration by promoting ubiquitination and degradation of vimentin, affecting cytoskeletal dynamics. In contrast, CD24 targets RCC2 for degradation, thereby regulating β-catenin signaling. Notably, RCC2 KO enhances β-catenin activity by suppressing inhibitors AXIN2 and APC, whereas CD24 KO inhibits this pathway. These findings reveal a regulatory loop where CD24 and RCC2 reciprocally control proliferation and metastasis, positioning the CD24-RCC2 axis as a promising therapeutic target in prostate cancer.

## Abstract 2

CD24 regulates RCC2 via ubiquitination, influencing β-catenin signaling. RCC2 suppresses metastasis by promoting cytoskeletal reorganization and vimentin degradation.

## Introduction

Prostate cancer continues to present substantial clinical challenges due to its intrinsic biological heterogeneity and diverse mechanisms of progression (1, 2). Understanding the molecular drivers of prostate cancer progression is critical for developing targeted therapeutic strategies. One molecule increasingly implicated in prostate cancer pathogenesis is CD24 (3–7), a glycosyl-phosphatidyl-inositol–anchored (GPI-anchored) protein. Although minimally expressed in healthy prostate epithelial cells, CD24 is markedly elevated in approximately half of prostate cancer cases, correlating strongly with increased metastatic potential and poor clinical outcomes (3, 4, 6–8). Our previous studies have shown that CD24 can promote tumor growth by inhibiting the ARF-NPM interaction, leading to ARF degradation, elevated MDM2 levels, and subsequent downregulation of p53-target genes (6, 7). However, the precise molecular mechanisms through which CD24 contributes specifically to prostate cancer metastasis remain poorly characterized.

Early studies proposed that CD24-mediated tumor metastasis might occur through its binding to P- and E-selectin (9, 10), and it has also been suggested to influence various pathways related to cellular motility, adhesion, and growth. However, the definitive causal relationship between CD24 overexpression and metastasis has not been fully established. Recently, CD24 has emerged as a pivotal regulator of metastasis, metabolism, and therapy resistance through diverse mechanisms: activating Arf6-ERK in esophageal cancer (11), reprogramming mitochondrial metabolism in breast cancer (12), inducing chemoresistance via miRNAs in ovarian cancer (13), and enabling immune evasion through Siglec-10 interactions (14). While CD24 overexpression correlates with metastasis across cancers, its direct causal role remains unclear.

Regulator of Chromosome Condensation 2 (RCC2), also known as TD-60, is an evolutionarily conserved multifunctional protein implicated in numerous cellular processes, including mitotic progression and cell migration (15). RCC2 is frequently overexpressed in various cancers, where it is associated with tumor aggressiveness and poor prognosis (16–18). Mechanistically, RCC2 influences cell motility through interactions with focal adhesion complexes and modulation of small GTPases, such as RAC1 and ARF6, which play critical roles in regulating directional migration and cytoskeletal dynamics (19). However, RCC2 has diverse roles in different cancers. In prostate cancer, RCC2 promotes cell proliferation and migration through the Hedgehog/GLI1 pathway (15). In lung, breast, and gastric cancers, RCC2 boosts cell motility and metastasis via epithelial-mesenchymal transition (EMT) (20–22). Conversely, RCC2 as a p53 target is involved in the suppression of metastasis in colorectal cancer (23). Therefore, conflicting evidence exists regarding the role of RCC2 in metastasis across different tumor types and experimental contexts, emphasizing the need for more detailed studies to clarify RCC2 function in prostate cancer progression and metastasis.

In this context, our study provides the first evidence of a direct interaction between CD24 and RCC2 in prostate cancer cells. focusing on their combined roles in prostate cancer proliferation and metastasis. Utilizing comprehensive molecular, cellular, and in vivo approaches, we uncovered a complex reciprocal regulatory mechanism involving ubiquitination and proteasomal degradation that specifically modulates the β-catenin signaling pathway. By elucidating this previously unknown interaction and its downstream effects, we addressed gaps in the understanding of how CD24 and RCC2 cooperatively control prostate cancer progression.

## Results

### CD24 is positively correlated with RCC2 in human prostate cancer.

**Figure 1:**
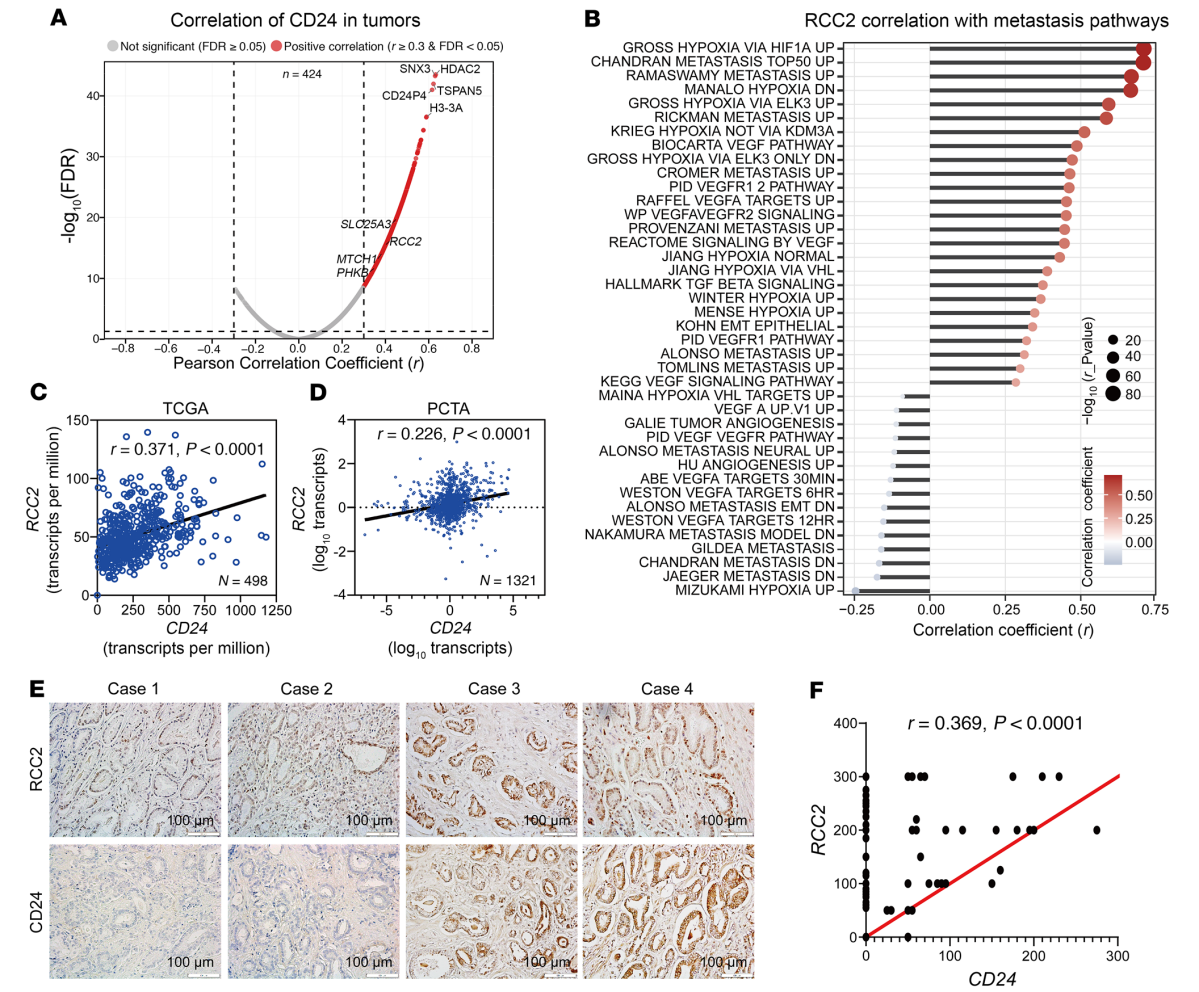
Positive correlation between CD24 and RCC2 expression in human prostate cancer tissues. (**A**) Volcano plot showing the correlation between *CD24* and other transcriptome genes in human prostate adenocarcinoma tissues (TCGA dataset, *n* = 424). The *x*-axis represents the Pearson correlation coefficient (*r*), and the *y*-axis displays the –log_10_(FDR). Gray dots indicate genes with nonsignificant correlations (FDR ≥ 0.05), while red dots represent genes with a significant positive correlation (*r* > 0.30 and FDR < 0.05). (**B**) Correlation analysis between *RCC2* expression and metastasis-related pathway activity based on Gene Set Variation Analysis (GSVA) in the TCGA dataset (*n* = 550). The *x*-axis shows the *r*, and the *y*-axis lists metastasis-related pathways. Dot color represents the correlation value (blue, negative; red, positive), while dot size reflects statistical significance, with larger dots corresponding to smaller *P*-values (–log_10_(*P*)). (**C**) Scatter plot showing a moderate positive correlation between *CD24* and *RCC2* mRNA expression levels in human prostate adenocarcinoma tissues (TCGA dataset). (**D**) Scatter plot showing a weak to moderate correlation between CD24 and RCC2 mRNA expression levels in the Prostate Cancer Transcriptome Atlas (PCTA) dataset. (**E**) Representative IHC staining of CD24 and RCC2 in human prostate cancer samples. Scale bar: 100 μm. (**F**) H-score quantitative analysis of IHC data reveals a moderate positive correlation between CD24 and RCC2 protein expression levels in primary prostate cancer samples (*n* = 78). **A**–**D**, and **F**, *r* was determined using Pearson’s correlation test. The experiments were repeated twice.

CD24 plays a crucial role in tumor metastasis (24), including prostate cancer (3, 7). Although we previously established that intracellular CD24 stimulates prostate cancer cell growth by controlling the ARF-NPM interaction and p53 inactivation (7), the mechanism of CD24-mediated metastasis remains elusive. To identify potential mechanisms, particularly other genes that interact with CD24 to promote metastasis, we screened potential CD24-associated genes using 2 public datasets: the Cancer Genome Atlas Program (TCGA) and the Prostate Cancer Transcriptome Atlas (PCTA). As shown in Figure 1A, we employed a comprehensive data-driven approach to perform a bioinformatics analysis of public datasets for the identification of CD24-associated interactors in the TCGA dataset. First, we identified genes positively correlated with CD24 expression, including RCC2 (Pearson correlation coefficient (*r*) > 0.30, *P* < 0.001). Among several candidate CD24 interactor genes, RCC2 was significantly associated with metastasis-related pathways in human prostate cancer (Figure 1B). These analyses of expression correlation and metastasis pathway enrichment suggest that RCC2 is a key player in CD24-mediated metastasis. Furthermore, we identified a weak-to-moderate positive correlation between CD24 and RCC2 mRNA expression levels in human prostate cancer tissues from the TCGA (*r* = 0.371, *P* < 0.0001) and PCTA (*r* = 0.226, *P* < 0.0001) datasets (Figure 1, C and D, and Supplemental Figure 1, A and B; supplemental material available online with this article; https://doi.org/10.1172/JCI192883DS1). Although CD24 is known to be androgen regulated in prostate cancer cells (25), there was no correlation in the TCGA dataset between CD24 expression and androgen receptor (AR) expression, or between CD24 and the AR-regulated gene *KLK3* (prostate-specific antigen, PSA) (Supplemental Figure 1C). Similarly, although *RCC2* expression showed a weak correlation with *AR*, it did not correlate with *KLK3* expression (Supplemental Figure 1D). In the PCTA dataset, we also observed a weak correlation between *CD24* and *AR* expression, as well as between *CD24* and *KLK3* (*PSA*) expression (Supplemental Figure 1C). Likewise, *RCC2* showed a weak correlation with *AR* but no correlation with *KLK3* (*PSA*) (Supplemental Figure 1D). Additionally, CD24 mRNA expression was lowest in the hormone-sensitive prostate cancer cell line LNCaP, moderate in the AR-positive castration-resistant prostate cancer (CRPC) cell line 22RV1, and highest in AR-negative CRPC cell lines (DU145 and PC3) and neuroendocrine prostate cancer (NEPC) cell lines (H660 and VCaP) (Supplemental Figure 1E). In contrast, RCC2 mRNA expression did not significantly differ among hormone-sensitive prostate cancer, CRPC, or NEPC cell lines (Supplemental Figure 1F). These findings suggest that CD24, but not RCC2, may function in relation to AR signaling.

Furthermore, using IHC analysis, we evaluated the protein expression of CD24 and RCC2 in 78 primary prostate adenocarcinoma samples (Figure 1E). Approximately 49% (38 of 78) and 82% (64 of 78) of prostate cancer samples showed CD24 and RCC2 expression, respectively. Notably, H-score quantitative analysis revealed a moderate positive correlation between the protein expression levels of CD24 and RCC2 in primary prostate adenocarcinomas (*r* = 0.369, *P* < 0.0001; Figure 1F and Supplemental Table 1). RCC2 protein expression was significantly decreased only in Gleason score 8–10 samples compared to Gleason score 7 samples (*P* = 0.031) (Supplemental Figure 1G). However, no significant changes in RCC2 protein expression were observed across tumor stages (T2, T3, T4, or metastatic cases) (Supplemental Figure 1H).

**Figure 2:**
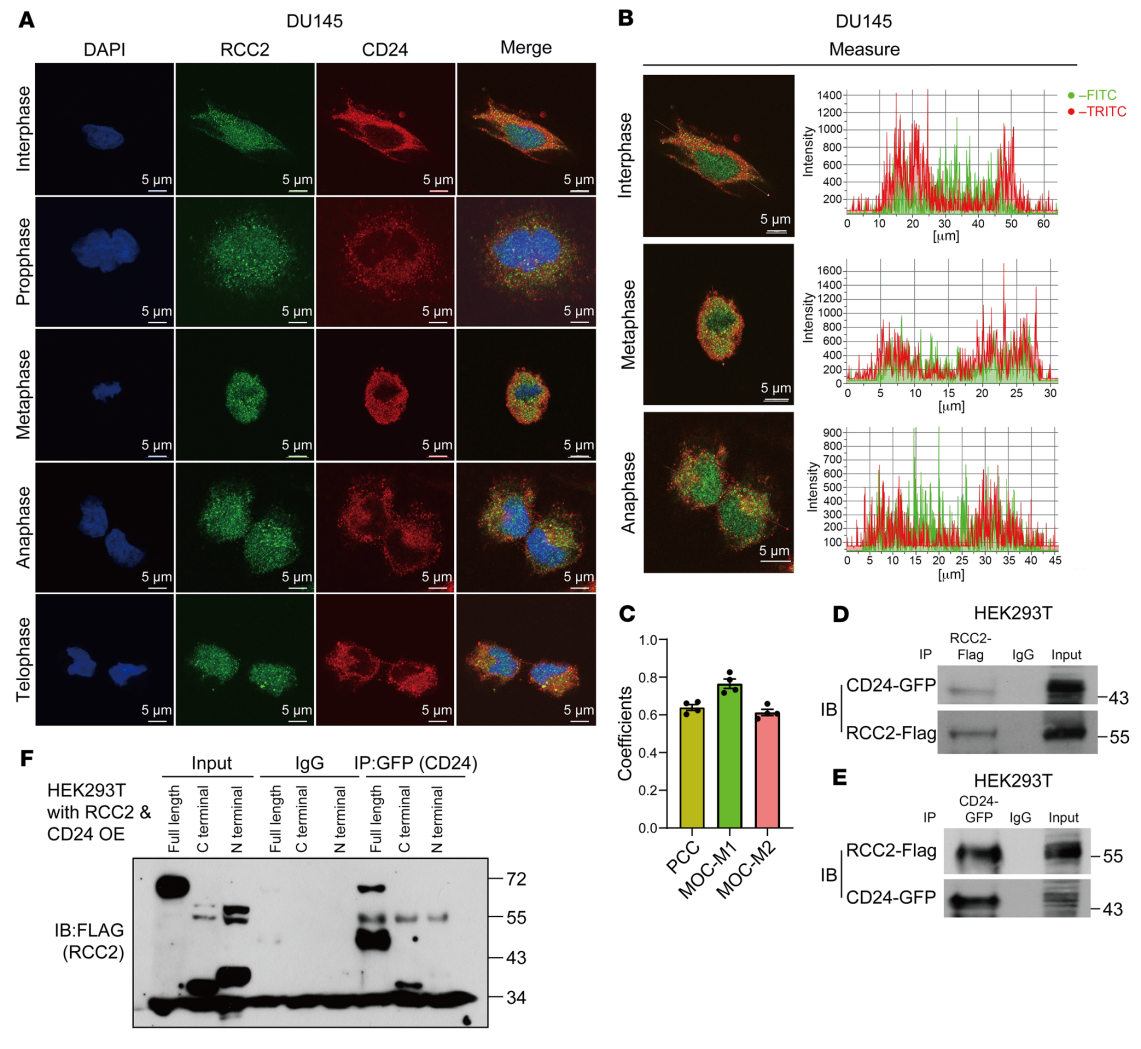
Colocalization and interaction of CD24 and RCC2 in DU145 cells. (**A**) Immunofluorescence images showing the localization of CD24 and RCC2 in DU145 cells during various cell cycle phases using specific anti-CD24 and anti-RCC2 antibodies. Scale bar: 5 μm. (**B**) Overlapping intensity patterns of CD24 and RCC2 were observed by analyzing pixel intensity values throughout the nucleus and cytoplasm. The images in **B** are derived from **A**, presenting overlapping intensity patterns via pixel intensity analysis. (**C**) Quantitative analysis of CD24 and RCC2 colocalization using ImageJ/Fiji with the JaCoP plugin. Pearson’s correlation coefficient (PCC) indicates the linear relationship between the fluorescence intensities of CD24 and RCC2. Manders’ overlap coefficient (MOC) values represent the degree of signal overlap: MOC-M1 reflects the fraction of CD24 signal overlapping with RCC2, while MOC-M2 reflects the fraction of RCC2 signal overlapping with CD24. Data were obtained from 3–4 independent immunofluorescence experiments, each with 3–4 images per condition (6–8 cells per image). Data are presented as mean ± SE. The coefficients were determined using Pearson’s correlation test. (**D** and **E**) Coimmunoprecipitation (co-IP) assays for the reciprocal binding between CD24 and RCC2 in transiently coexpressed HEK293T cells using specific anti-CD24 and anti-RCC2 antibodies. (**F**) Mapping of the binding regions between CD24 and RCC2 in HEK 293T cells overexpressing GFP-tagged full-length CD24 and Flag-tagged RCC2 domains. All experiments were repeated 3 times.

To determine whether RCC2 interacts with CD24 in prostate cancer cells, we analyzed the localization of CD24 and RCC2 in DU145 cells using immunofluorescence (IF). CD24 is a cell surface marker, but the majority reside intracellularly in DU145 cells (7). As shown in Figure 2A, RCC2 was expressed in both the nucleus and cytoplasm of DU145 cells during the interphase, whereas CD24 was localized in the cytoplasm. However, RCC2, which accumulates in the nucleus, is widely dispersed in the cytoplasm during mitosis, particularly during metaphase. CD24 and RCC2 colocalized in the cytosol of DU145 cells during interphase and mitosis. Notably, overlapping intensity patterns of RCC2 and CD24 were observed by analyzing the precise pixel intensity values throughout the nucleus and cytoplasm (Figure 2B). A quantitative colocalization analysis of CD24 and RCC2 was performed using ImageJ/Fiji with the JaCoP plugin to assess the relationship between the fluorescence intensities of the 2 proteins and to further quantify their colocalization. As shown in Figure 2C, there was strong colocalization between CD24 and RCC2, as indicated by Pearson’s correlation coefficient (PCC, *r* = 0.6393), Manders’ overlap coefficient (MOC)-M1 (CD24 vs. RCC2, *r* = 0.7655), and MOC- M2 (RCC2 vs. CD24, *r* = 0.6130).

Furthermore, we cotransfected *CD24* and *RCC2* into HEK293T cells to examine their potential interactions. In transiently overexpressing HEK293T cells, coimmunoprecipitation (co-IP) confirmed direct binding between CD24 and RCC2 (Figure 2, D and E). To map the binding regions between CD24 and RCC2, GFP-tagged full-length CD24 and Flag-tagged full-length RCC2 N- or C-terminal cDNA domain constructs were cointroduced to overexpress CD24 and RCC2 in HEK 293T cells. As shown in Figure 2F, both the C-terminal and N-terminal domains of RCC2 were immunoprecipitated using anti-GFP antibody incubation and identified using anti-FLAG antibody incubation. These data indicated that RCC2 and CD24, as interacting partners, are coexpressed in the cytosol of prostate cancer cells.

To evaluate *RCC2* expression and its clinical relevance in human prostate cancer, we conducted bioinformatics analysis of TCGA and PCTA datasets. According to TCGA dataset, *RCC2* expression was upregulated in most human cancer types (16 of 24), including prostate cancer (Supplemental Figure 2A). For prostate adenocarcinoma, *RCC2* expression was higher in tumor samples than in normal prostate samples and likely increased with Gleason score, tumor stage, and metastasis, but was not significantly related to patient overall survival (Supplemental Figure 2, B–F). However, high *RCC2* expression was likely associated with poor survival in patients with other cancers, including breast cancer, liver cancer, and mesothelioma (Supplemental Figure 2, G–I). Furthermore, we performed bioinformatics analysis using cBioPortal datasets to assess genetic alterations in *RCC2* among 10,998 human prostate cancer samples from 26 studies, including the TCGA dataset. Genetic alterations in *RCC2*, including amplification, deletion, and mutation, were found in only 1.2% of the samples.

### Establishment of CD24 and RCC2-KO prostate cancer cells.

In our previous studies, we analyzed the expression of *CD24* mRNA and protein in 3 human prostate cancer cell lines: DU145, PC3, and LNCaP (7). The metastatic CRPC cell lines DU145 and PC3 express high and low levels of CD24, respectively, whereas androgen-dependent LNCaP cells do not express detectable amounts of CD24 (7). Similarly, we determined the expression levels of RCC2 in the 3 prostate cancer cell lines. DU145 cells expressed the highest levels of RCC2, whereas LNCaP cells expressed the lowest levels among the 3 cell lines (Supplemental Figure 3A). Using CRISPR/Cas9 genome editing with 2 distinct single-guide RNAs (sgRNAs), we developed *CD24*-and/or *RCC2* KO CRPC cell models. As shown in Supplemental Figure 3, B and C, we established *CD24-*KO DU145 cell lines (2 clones) and *RCC2-*KO DU145 cell lines (2 clones). These cell lines were validated by Sanger sequencing, Western blotting, and flow cytometry (Supplemental Figure 3, B, C, E, and F, and Supplemental Figure 4A). PC3 cells express low levels of CD24 in their cytoplasm (7). As shown in Supplemental Figure 3D and Supplemental Figure 5A, we established *RCC2-*KO PC3 cell lines (2 clones), which were validated by DNA sequencing and Western blotting. Finally, all selected KO colonies were analyzed using the Cas-OFFinder web tool to predict the potential off-target regions of *CD24* and *RCC2* sgRNAs (Supplemental Figure 3, G and H), as described previously (26, 27). Notably, 4 nucleotide-mismatched genes, *MARS*, *MAPKAPK3*, *CPNE2,* and *PRKCD,* were predicted to be potential off-targets for *RCC2* sgRNAs. Denaturing gradient gel electrophoresis was performed to exclude potential off-target effects on these genes (Supplemental Figure 3I).

### Effect of CD24 and RCC2 on prostate tumor growth and metastasis.

**Figure 3:**
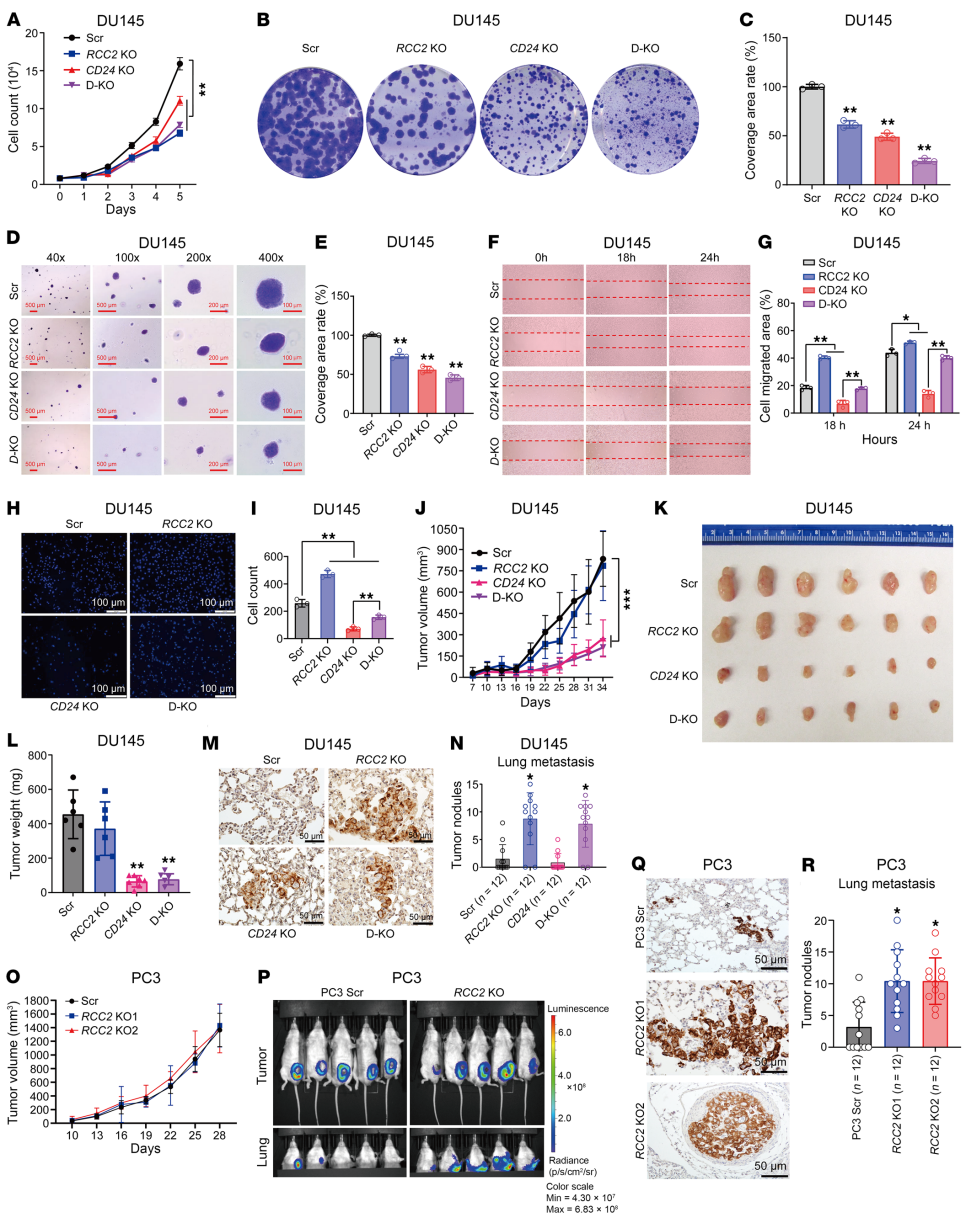
Effects of *CD24* and *RCC2* knockouts on cell proliferation, migration, tumor growth, and metastasis in human prostate cancer cell models. (**A**–**E**) Cell proliferation assays in *CD24* knockout (KO), *RCC2* KO, and *CD24/RCC2* KO (D-KO) DU145 cells compared to scrambled control (Scr) cells, evaluated by cell growth, colony formation, and soft agar assays. Scale bars (**D**): 500 μm (40× and 100× panels); 200 μm (200× panel); and 100 μm (400× panel) (**F**–**I**) Cell migration rates determined by wound healing and Transwell migration assays in *CD24* KO, *RCC2* KO, and *CD24/RCC2* KO DU145 cells compared with scrambled control cells. Red dotted lines indicate the edge of cell migration. Blue DAPI staining dots refer to the cells that have crossed the transwell chamber membrane. Scale bar (**H**): 100 μm. (**J**–**L**) In vivo tumor growth analysis in NSG mice subcutaneously inoculated with DU145 cells, showing tumor volumes, sizes, and weights in *CD24* KO, *RCC2* KO, and *CD24/RCC2* KO xenografts compared with scrambled controls. (**M** and **N**) Lung metastasis rates and gross tumor nodules in *CD24* KO, *RCC2* KO, and *CD24/RCC2* KO xenografts compared with scrambled controls, determined by IHC analysis with a specific anti-human vimentin antibody, staining shows lung metastatic tumor cells with Vimentin expression. Scale bar (**M**): 50 μm. (**O** and **P**) Tumor growth, weight, and lung metastasis in NSG mice implanted with luciferase-labeled PC3 cells with or without *RCC2* KO. (**Q** and **R**) Quantitative analysis of lung metastasis in PC3 xenografts in *RCC2* KO compared to scrambled controls by IHC analysis with a specific anti-human vimentin antibody, staining shows lung metastatic tumor cells with Vimentin expression. Scale bars (**Q**): 50 μm. Data are presented as mean ± SD. *P < 0.05, **P < 0.01, and ***P < 0.001 by 1-way ANOVA with Tukey’s multiple comparisons test (**C**, **G**, **I**, **L**, **N**, and **R**) or 2-way ANOVA vs. scrambled control group (**A**, **J**, and **O**). All experiments were repeated 2 or 3 times.

Our previous studies demonstrated the oncogenic role of CD24 in tumor growth and metastasis in prostate cancer (7). Notably, targeted mutation and short hairpin RNA (shRNA) silencing of *CD24* reduced cell proliferation and migration and retarded xenograft tumor growth, progression, and lung metastasis. Next, we tested the effect of *RCC2* KO on the biological activities of DU145 and PC3 cells. In vitro analysis showed an inhibitory effect of *RCC2* KO on the proliferation of DU145 and PC3 cells, as evidenced by cell growth, colony formation, and soft agar assays (Supplemental Figure 4, A–E, and Supplemental Figure 5, A–E). However, *RCC2* KO promoted the migratory capacity of DU145 and PC3 cells, as demonstrated by the wound healing and transwell migration assays (Supplemental Figure 4, F–I, and Supplemental Figure 5, F–I). To determine whether the effect of *RCC2* KO on cell proliferation and migration was related to CD24 expression, we established *CD24* and *RCC2* double-KO (*CD24*/*RCC2* KO) DU145 cells. Using the established DU145 cell model, we assessed the role of CD24, RCC2, and their interaction in tumor cell proliferation and migration. As shown in Figure 3, A–E, *RCC2* KO, *CD24* KO, and *CD24*/*RCC2* KO cells exhibited decreased cell proliferation compared with scrambled control cells, as measured by cell growth, colony formation, and soft agar assays. Notably, DU145 cells with *RCC2* KO migrated faster, while *CD24* KO or *CD24*/*RCC2* KO migrated slower than scrambled control cells in wound healing and transwell migration (Figure 3, F–I). Cell proliferation was reduced in *CD24*/*RCC2* KO cells compared with proliferation in other cells, suggesting that dual targeting *CD24* and *RCC2* effectively inhibits cell proliferation in DU145 cells. However, cell migration was increased in *RCC2*-KO cells but decreased in *CD24*-KO cells compared with scrambled control cells. Collectively, these findings suggest a synergistic role of CD24 and RCC2 in promoting cell proliferation but opposing functions in regulating cell migration.

To further investigate the functions of CD24 and RCC2 in vivo, we subcutaneously inoculated scrambled control, *RCC2* KO, *CD24* KO, and *CD24*/*RCC2* KO DU145 cells into the lower left quadrant of the abdomen of 6-week-old male NOD SCID gamma (NSG) mice and monitored the tumor growth and spontaneous metastasis. As shown in Figure 3, J–L, tumor volume, size, and weight were reduced in *CD24*-KO and *CD24*/*RCC2-*KO xenograft mice compared with those in scrambled control xenograft mice. However, there was no significant difference in tumor volume, size, or weight between *RCC2*-KO and scrambled control xenograft mice. This in vivo result is in contrast to the in vitro observation of cell growth differences between *RCC2*-KO and scrambled control cells. Additionally, there was no significant difference in tumor volume, size, or weight between *CD24*-KO and *CD24*/*RCC2-*KO xenograft mice. Eight weeks after injection, lung metastasis was assessed by IHC analysis using an antivimentin antibody in all 4 animal groups. Ten out of 12 mice in the *RCC2*-KO and *CD24/RCC2*-KO xenograft groups developed spontaneous lung metastases, compared with fewer mice in the scrambled control (5 of 12) and *CD24*-KO (3 of 12) groups. Quantitative analysis revealed approximately 10-fold more gross tumor nodules in the lungs of *RCC2*-KO and *CD24*/*RCC2-*KO xenograft mice than in scrambled control and *CD24*-KO xenograft mice (Figure 3, M and N). These data suggest that RCC2 is more likely to be involved in regulating tumor metastasis than in promoting tumor growth in prostate cancer. To validate the role of RCC2 in tumor metastasis in vivo, we used an additional metastatic CRPC cell line, the transgenic luciferase-labeled PC3 cell line, with or without *RCC2* implanted subcutaneously into 6-week-old male NSG mice. Mice bearing scrambled control and *RCC2*-KO PC3 cells showed similar tumor growth (Figure 3O). At 8 weeks post injection, lung metastasis was detected in both scrambled control (8 of 12) and *RCC2*-KO (12 of 12) groups. Quantitative analysis indicated more than a 3-fold increase in gross tumor nodules in the lungs of *RCC2*-KO xenograft mice compared with scrambled control xenograft mice (Figure 3, P–R). Collectively, these data suggest that *RCC2* KO induces spontaneous lung metastasis in prostate cancer cells.

**Figure 4:**
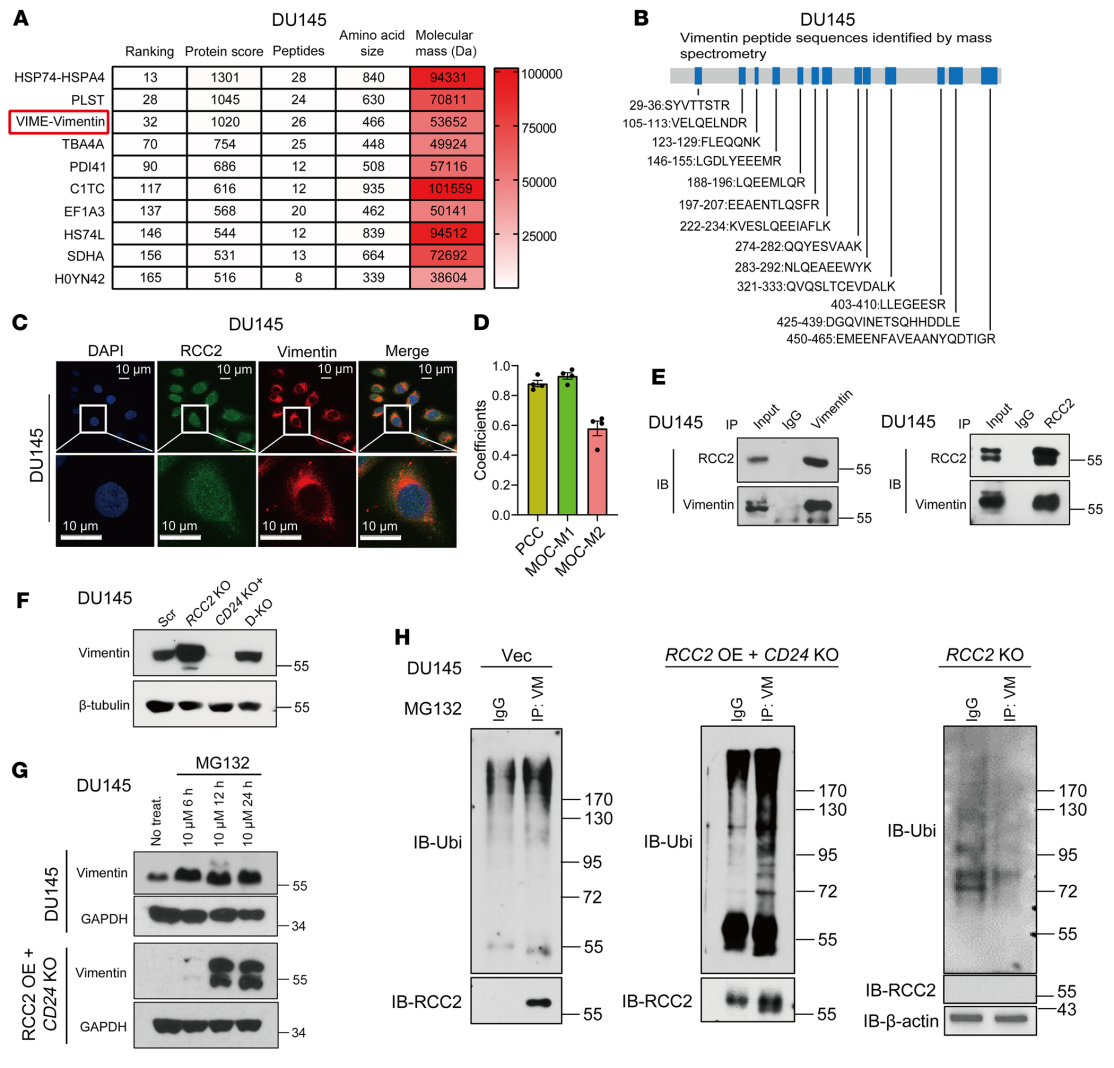
Interaction between RCC2 and Vimentin and the role of RCC2 in Vimentin degradation in DU145 cells. (**A**) Identification of Vimentin as a top RCC2-binding partner by mass spectrometry analysis of Flag-RCC2 overexpressed in HEK293T cells, showing 13 matching peptides from Vimentin. (**B**) Table listing Vimentin peptides identified by mass spectrometry, highlighting the potential interaction sites with RCC2. (**C**) Immunofluorescence images showing the localization of RCC2 and Vimentin in DU145 cells using specific anti-RCC2 and anti-Vimentin antibodies. Scale bar: 10 μm. (**D**) Quantitative analysis of RCC2 and vimentin colocalization using ImageJ/Fiji with the JaCoP plugin. Pearson’s correlation coefficient (PCC) indicates the linear relationship between the fluorescence intensities of RCC2 and vimentin. Manders’ overlap coefficient (MOC) values represent the extent of signal overlap: MOC-M1 reflects the fraction of RCC2 signal overlapping with vimentin, while MOC-M2 reflects the fraction of vimentin signal overlapping with RCC2. Data were obtained from 3–4 independent immunofluorescence experiments, each with 3–4 images per condition (6–8 cells per image). Data are presented as mean ± SE. The coefficients were determined using Pearson’s correlation test. (**E**) Coimmunoprecipitation (co-IP) assays with anti-RCC2 and anti-Vimentin antibodies in DU145 cells confirming the interaction between RCC2 and Vimentin. (**F**) Immunoblot analysis showing Vimentin expression in *RCC2* KO cells, *CD24* KO + *RCC2* overexpression (OE) cells, and *CD24/RCC2* double KO (D-KO) cells. (**G**) Vimentin expression in scrambled control and *RCC2* OE + *CD24* KO DU145 cells treated with the proteasome inhibitor MG-132 (10 μM) for 6, 12, and 24 hours. (**H**) Immunoprecipitation with anti-Vimentin followed by anti-Ubiquitin immunoblotting in MG-132-treated lysates of scrambled control, *RCC2* OE + *CD24* KO, and *RCC2* KO DU145 cells. The experiments were repeated 3 times.

To elucidate the molecular mechanism of *RCC2*-KO–induced cell migration, we performed mass spectrometry analysis using a Flag-RCC2 construct or an empty vector overexpressed in HEK293T cells to identify the potential RCC2 binding partners (Supplemental Table 2). Notably, Vimentin was identified as one of the top 3 RCC2 binding partners with 13 matching peptides (Figure 4, A and B). Vimentin is the most widely expressed intermediate filament protein component and undergoes dramatic reorganization during mesenchymal and cancer cell migration (28). To validate the interaction between Vimentin and RCC2, we performed IF and coIP assays using either anti-RCC2 or anti-vimentin antibodies in DU145 cells, which confirmed that Vimentin and RCC2 interact (Figure 4, C–E). Next, we transfected a vector containing the full-length RCC2 coding sequence into *CD24*-KO DU145 cells to create cells with overexpressed *RCC2* and *CD24* KO (*CD24* KO+*RCC2* overexpression [OE]). In *RCC2*-KO cells, vimentin expression increased but disappeared when *RCC2* was overexpressed in *CD24*-KO cells, and vimentin expression was restored when *RCC2* was knocked out in *CD24*-KO cells (Figure 4F). Vimentin levels are regulated by the ubiquitin-proteasome system (29, 30). To examine whether RCC2 degrades vimentin via ubiquitination, we treated scrambled control and *RCC2*-OE + *CD24*-KO DU145 cells with the proteasome inhibitor MG-132 for 6, 12, and 24 hours at a dose of 10 μM. MG-132 treatment for 12 hours and 24 hours stabilized vimentin protein expression in DU145 cells (Figure 4G). Notably, Vimentin protein stabilization was more pronounced when RCC2 was overexpressed in *CD24*-KO DU145 cells after MG-132 treatment (Figure 4G), suggesting that RCC2 may regulate vimentin protein expression through ubiquitin-mediated degradation. Subsequently, we performed immunoprecipitation with an anti-vimentin antibody, followed by immunoblotting with an anti-ubiquitin antibody, using MG-132 treated lysates from parental DU145 cells, *RCC2*-OE + *CD24*-KO DU145 cells, and *RCC2-*KO DU145 cells. The results showed that *RCC2* overexpression markedly enhanced vimentin ubiquitination, whereas *RCC2* KO reduced vimentin ubiquitination compared with parental DU145 cells (Figure 4H). *VIM* is a transcriptional target of β-catenin. In TCGA dataset, we identified a negative correlation between *RCC2* and *VIM* mRNA expression levels (*r* = –0.160, *P* < 0.001) in primary prostate cancer tissues (Supplemental Figure 6A). Furthermore, IHC analysis of vimentin protein expression in primary prostate cancer samples revealed a negative correlation between RCC2 and Vimentin protein expression levels (*r* = –0.464, *P* < 0.001) (Supplemental Figure 6, B and C).

Intermediate filaments interact with microfilaments and microtubules to regulate the cell cytoskeleton, thus influencing directed cell migration (31, 32). To further assess the effects of *CD24* and/or *RCC2* KO on microfilament and microtubule organization, we stained the cells with F-actin and α-tubulin antibodies for IF analysis. Compared with scrambled control DU145 cells, F-actin filaments appeared compressed throughout the cytoplasm in *RCC2*-KO cells but were loosely distributed in *CD24*-KO cells (Supplemental Figure 7A). Notably, *RCC2*-KO cells had compacted F-actin filaments that were otherwise unconsolidated in *CD24*-KO cells. Additionally, α-tubulin staining showed that microtubules in *RCC2*-KO DU145 cells were sparse and condensed compared with those in the scrambled control cells (Supplemental Figure 7B). In *CD24*-KO cells, the microtubules were short and perinuclear, whereas in *CD24/RCC2*-KO cells, they were elongated and expanded (Supplemental Figure 7B). Collectively, these data indicated that CD24 and RCC2 may interact to regulate the cytoskeleton in prostate cancer cells.

One essential function of RCC2 is the attenuation of fibronectin-induced (FN-induced) activation of small GTPases RAC1 and ARF6, which regulate directional cell migration (33). RAC1 is a critical regulator of mesenchymal-like migration, axonal growth, and cell adhesion, and its activation correlates with aggressive malignant characteristics, such as tumor invasion and metastasis, in several tumor types. ARF6 is a critical mediator of endocytosis and membrane recycling at the cell surface, and ARF6 activation promotes invasion and metastasis of various cancer cells. As determined by binding assays with downstream effector proteins of GTP-bound RAC1 and ARF6, cell adhesion to a FN substrate can induce activation of RAC1 and ARF6 during cell spreading (33). We investigated the effect of *RCC2* KO on the FN-dependent activation of RAC1 and ARF6 in prostate cancer cells. As shown in Supplemental Figure 7C, after FN stimulation, GTP-RAC1 expression levels peaked at 10 minutes, decreased at 30–60 minutes, and increased again at 90 minutes, showing similar activation patterns in both the scrambled control and *RCC2*-KO cells. Furthermore, after FN stimulation, maximal levels of GTP-ARF6 expression were observed at 10 minutes, which decreased from 30 to 120 minutes in scrambled control cells, whereas GTP-ARF6 levels decreased from 15 to 30 minutes and increased again from 60 to 120 minutes in *RCC2*-KO cells (Supplemental Figure 7D). These data suggested that RCC2 is unlikely to be responsible for FN-dependent RAC1 and ARF6 activation and related cell migration in DU145 cells.

### Prostate-specific deletion of Rcc2 delays tumor development but promotes tumor metastasis in prostate cancer mouse models.

**Figure 5:**
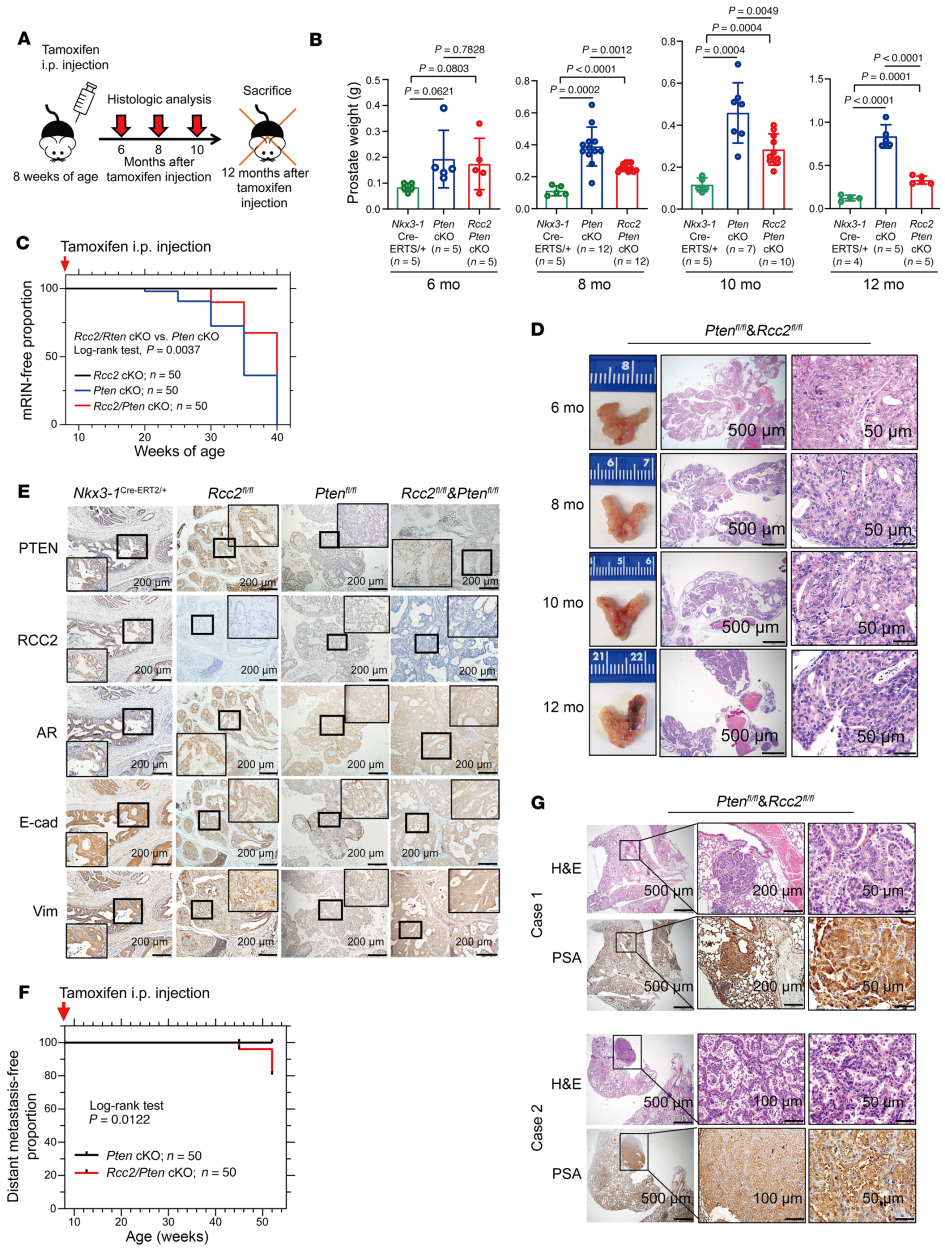
Role of *Rcc2* in spontaneous prostate cancer progression and metastasis in mouse models. (**A**) Schematic diagram of spontaneously developed prostate tumors followed up to 12 months of age in genetically engineered mouse models. Tamoxifen was administered at 8 weeks of age to induce Cre-mediated recombination in prostate epithelial cells. (**B**) Prostate weight analysis in *Rcc2*-cKO, *Pten*-cKO, and *Rcc2/Pten*-cKO mice compared with scrambled control mice at 6-, 8-, 10-, and 12-months after tamoxifen treatment. (**C**) Kaplan–Meier curves of mPIN incidences up to 40 weeks of age. At 20, 25, 30, 35, and 40 weeks of age, 5 mice per time point were sacrificed for pathological analysis. (**D**) Histological analysis of prostate tissues in *Rcc2/Pten*-cKO mice up to 12 months after tamoxifen treatment. Scale bars: 500 μm (left), 50 μm (right). (**E**) IHC staining in mouse prostate tissues with anti-mouse PTEN, RCC2, AR, E-cadherin, and Vimentin antibodies in *Rcc2*-cKO, *Pten*-cKO, and *Rcc2/Pten*-cKO mice compared with scrambled control mice at 6 months after tamoxifen treatment. Scale bars: 200 μm. (**F**) Incidence of lung metastasis in *Rcc2/Pten*-cKO mice compared with *Pten*-cKO mice at 12 months after tamoxifen treatment. (**G**) Representative IHC staining of lung metastatic lesions with anti-PSA antibody, confirming the prostatic origin of metastatic tumors. Data are presented as means ± SD. Scale bars in Case 1: 500 μm (left); 200 μm (middle); 50 μm (right). Scale bars in Case 2: 500 μm (left); 100 μm (middle); 50 μm (right). (**B**) *P* values were determined by 1-way ANOVA with Tukey’s multiple comparisons test. (**C** and **F**) The log-rank test was used to analyze tumor development or metastasis and compare the distribution of time to event between groups. AR, androgen receptor; cKO, conditional knockout; mPIN, mouse prostatic intraepithelial neoplasia; i.p., intraperitoneal injection; PSA, prostate-specific antigen. All experiments were repeated twice.

To validate the role of *Rcc2* in spontaneous prostate cancer, we crossed *Nkx3-1^CreERT2^* knock-in mice with *Rcc2-*floxed mice and/or *Pten-*floxed mice to create prostate conditional *Rcc2*-KO (*Rcc2-*cKO), *Pten-*cKO, and *Rcc2* and *Pten* double cKOs (*Rcc2/Pten-*cKO) mice on a C57BL/6 background (Supplemental Figure 8, A and B). All mice were treated with tamoxifen at 8 weeks of age to induce Cre recombinase (CreERT2) expression at the *Nkx3.1* locus in mouse prostate epithelial cells (Figure 5A). However, no histological changes were observed in the prostate of *Rcc2-*cKO mice compared with *Nkx3-1^CreERT2/+^* control mice for up to 12 months after tamoxifen treatment. Approximately 100% of *Pten-*cKO mice developed high-grade mouse prostatic neoplasia hyperplasia (mPIN) but no carcinoma or metastasis for up to 12 months (34). In *Rcc2/Pten*-cKO mice, prostate weight did not significantly change at 6 months after tamoxifen treatment but was reduced after 8 months, especially at 12 months, compared with *Pten*-cKO mice (Figure 5B), suggesting that *Rcc2/Pten*-cKO mice experienced slower prostate growth than *Pten*-cKO mice. Histological assessment revealed delayed formation of mPIN in *Rcc2/Pten-*cKO mice compared with *Pten-*cKO mice, but no invasion through the basement membrane was observed for up to 12 months (Figure 5, C and D). All mPIN lesions in the mice were androgen receptor–positive (AR-positive), with no changes in AR expression among groups (Figure 5E). Notably, *Rcc2/Pten*-cKO mPIN lesions showed increased vimentin expression and decreased E-cadherin expression compared with *Pten*-cKO lesions (Figure 5E). We also assessed the incidence of distant metastasis in various organs, including the lungs, liver, bone, and brain using histological analysis. Twelve months after tamoxifen treatment, 12% (6 of 50) of *Rcc2/Pten*-cKO mice developed lung metastasis, whereas none of the *Pten*-cKO mice developed distant metastasis (Figure 5F). IHC staining confirmed that all metastatic lung tumors were prostate-specific antigen–positive (Figure 5G). These data suggest that *Rcc2*-cKO facilitates prostate cancer metastasis to the lungs of *Pten*-cKO mice.

To further validate the role of *Rcc2* in spontaneous tumor metastasis, we crossed *Rcc2* cKO alleles with transgenic adenocarcinoma of the mouse prostate (TRAMP) mice on a C57BL/6 background (Supplemental Figure 8C). TRAMP mice express the *SV40* large T antigen, a potent oncogene, under the control of a prostate-specific rat probasin (PB) promoter and develop spontaneous prostate cancers with tumor metastasis. As shown in Supplemental Figure 9, A and B, the onset of prostate tumors was delayed in homozygous *Rcc2-*cKO TRAMP mice compared with the onset in heterozygous *Rcc2-*cKO or *Rcc2* WT TRAMP mice. However, homozygous *Rcc2*-cKO TRAMP mice died earlier than heterozygous *Rcc2*-cKO or *Rcc2* WT TRAMP mice (Supplemental Figure 9C). At 7 months of age, prostate weights were notably increased in heterozygous *Rcc2*-cKO and *Rcc2* WT TRAMP mice, but decreased in homozygous *Rcc2*-cKO mice (Supplemental Figure 9D). TRAMP mice develop lymphatic and lung metastases at 6 months of age. As summarized in Supplemental Figure 9, E–G, by 7 months, lung metastases were observed in 6.7% (2 of 30) of *Rcc2* WT, 10% (3 of 30) of heterozygous *Rcc2*-cKO, and 20% (6 of 30) of homozygous *Rcc2*-cKO TRAMP mice. IHC analysis revealed AR-positive prostate tumors in all groups, with lower E-cadherin and higher vimentin expression in *Rcc2*-cKO TRAMP tumors than in WT TRAMP tumors (Supplemental Figure 9H). These data suggest that prostate-specific inactivation of both *Rcc2* alleles reduces tumor growth but promotes metastasis.

### CD24 ubiquitinates and degrades RCC2 and cooperatively regulates the β-catenin signaling pathway in prostate cancer cells.

**Figure 6:**
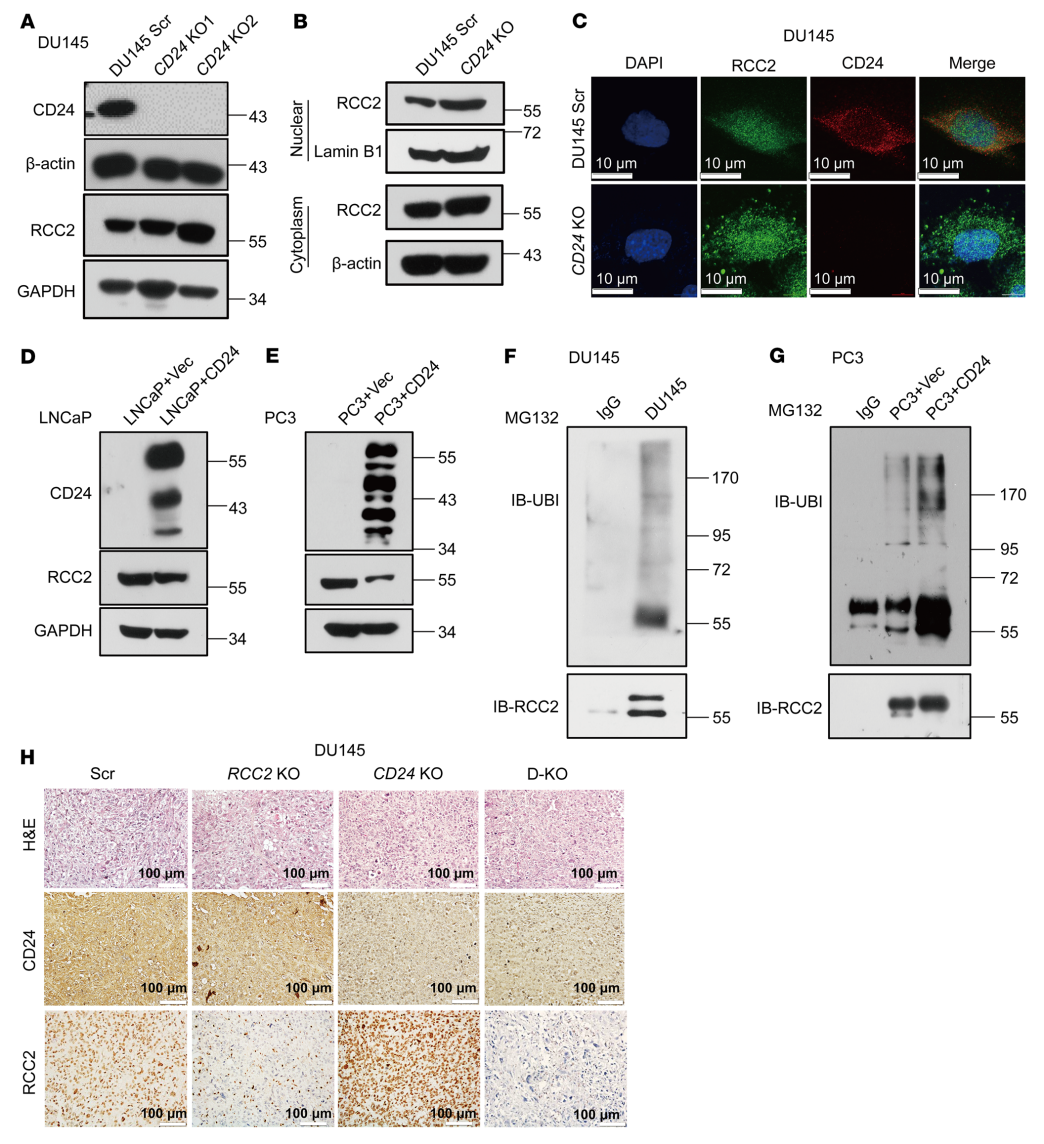
Regulation of RCC2 by CD24 and its impact on β-catenin signaling in prostate cancer cells. (**A**) Western blot analysis showing RCC2 expression in *CD24* knockout (KO) DU145 cells compared with scrambled control (Scr) cells. (**B**) Subcellular fractionation and Western blot analysis of RCC2 expression in the cytoplasm of *CD24* KO DU145 cells compared with Scr cells. (**C**) Immunofluorescence staining showing cytoplasmic accumulation of RCC2 in *CD24* KO DU145 cells relative to Scr cells. Scale bars: 10 μm. (**D** and **E**) Western blot analysis showing expression of CD24 and RCC2 in LNCaP and PC3 cells. (**F** and **G**) Western blot and immunoprecipitation analysis following MG-132 treatment (10 μM for 12 hours) showing stabilization of RCC2 protein expression and ubiquitination in endogenous CD24-expressing DU145 cells, empty vector-transduced PC3 cells, and *CD24*-overexpressing (*CD24* OE) PC3 cells. (**H**) IHC analysis of xenograft tumors from DU145 cells showing expression of CD24 and RCC2 in *CD24* KO, *RCC2* KO, and *CD24/RCC2* KO (D-KO) tumors compared to scrambled controls. Scale bars: 100 μm.

In DU145 cells, we observed that *CD24* KO overexpressed RCC2 (Figure 6A). Further analysis of the nuclear and cytoplasmic protein fractions revealed that RCC2 was predominantly overexpressed in the cytoplasm of *CD24*-KO cells (Figure 6B), whereas the level of CD24 expression was unaffected by *RCC2* KO (Supplemental Figure 3E). Likewise, IF staining showed an accumulation of RCC2 in the cytoplasm of *CD24*-KO cells compared with scrambled control cells (Figure 6C). We also transduced *CD24* into LNCaP and PC3 cells, and CD24 overexpression reduced RCC2 expression (Figure 6, D and E). To determine whether CD24 degrades RCC2 via ubiquitination, we treated endogenous CD24-expressiing DU145 cells, empty vector–transduced PC3 cells, and CD24-overexpressed PC3 cells with MG-132 (10 μM) for 12 hours. MG-132 treatment stabilized RCC2 protein expression in all treated cells (Figure 6, F and G). Subsequent immunoprecipitation with an anti-RCC2 antibody, followed by immunoblotting with an anti-ubiquitin antibody using MG-132-treated lysates, showed that CD24, particularly overexpression of CD24, markedly enhanced RCC2 ubiquitination in these cells (Figure 6, F and G). This indicates that CD24 ubiquitinates and degrades RCC2 protein in prostate cancer cells. IHC analysis further validated the overexpression of RCC2 in DU145 *CD24*-KO xenograft tumors (Figure 6H).

**Figure 7:**
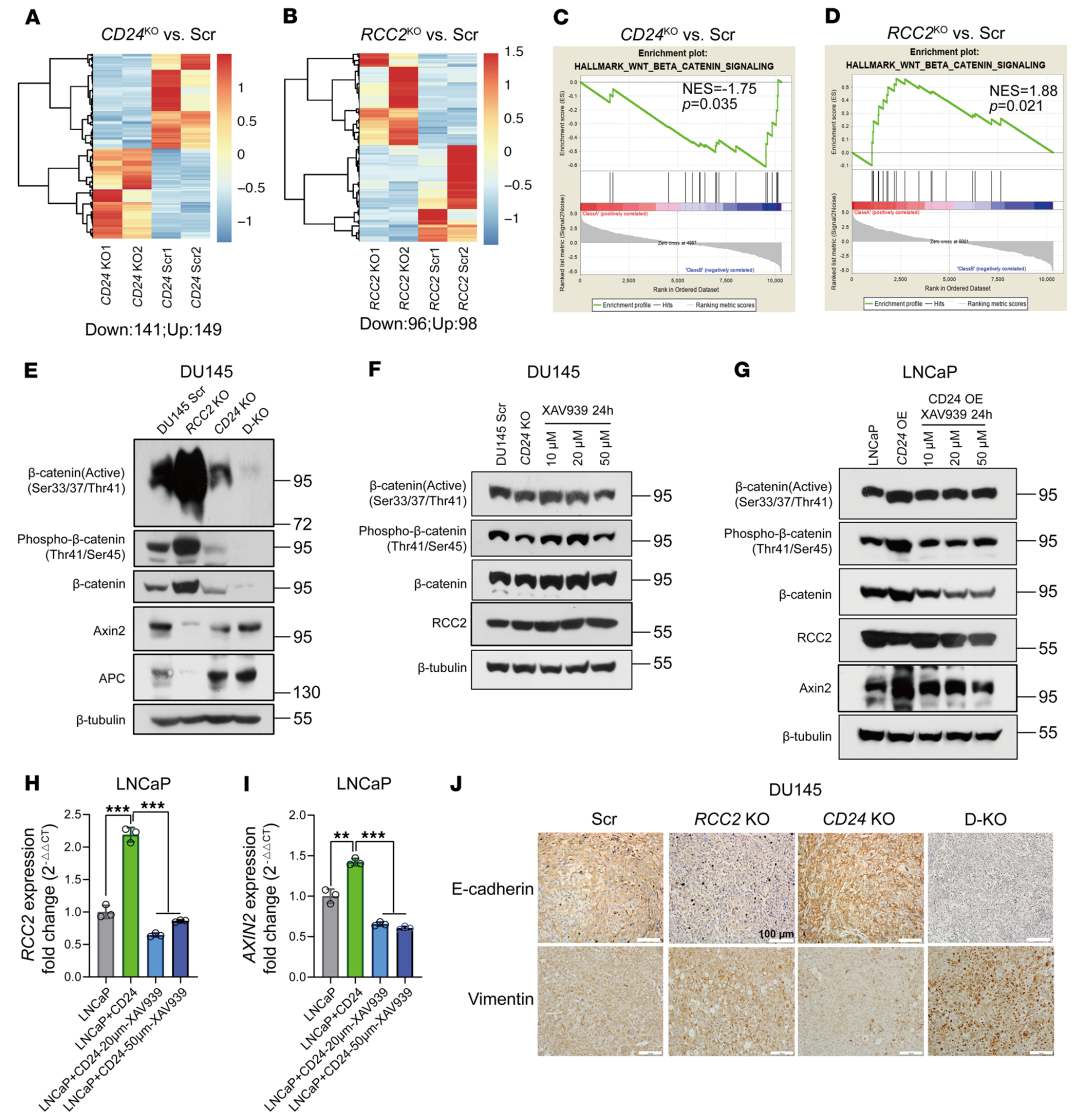
Effect of CD24 and RCC2 interaction on β-catenin signaling activation in prostate cancer cells. (**A** and **B**) Heatmaps of differentially expressed genes (DEGs) identified by RNA sequencing (RNA-Seq) in *CD24* knockout (KO) and *RCC2* KO DU145 cells compared to scrambled control cells. (**C** and **D**) Gene set enrichment analysis (GSEA) showing the enrichment of DEGs in the Wnt/β-catenin signaling pathway in *CD24* KO and *RCC2* KO cells compared to scrambled control cells. (**E**) Western blot analysis showing β-catenin activation and expression levels of Axin2 and APC in *CD24* KO, *RCC2* KO, and *CD24/RCC2* KO (D-KO) DU145 cells compared to scrambled cells. (**F**) Western blot analysis showing β-catenin activation and expression levels of RCC2 in *CD24* KO DU145 cells and XAV939-treated DU145 cells. (**G**) Western blot analysis showing β-catenin activation and expression levels of RCC2 and Axin2 in parental LNCaP cells, *CD24* OE LNCaP cells, and XAV939-treated *CD24* OE LNCaP cells. (**H** and **I**) mRNA expression levels of RCC2 and AXIN2 in parental LNCaP cells, *CD24* OE LNCaP cells, and XAV939-treated *CD24* OE LNCaP cells. (**J**) IHC analysis of xenograft tumors from DU145 cells showing expression of E-cadherin and Vimentin in *CD24* KO, *RCC2* KO, and *CD24/RCC2* KO (D-KO) tumors compared with scrambled controls. Scale bars: 100 μm. Data are presented as means ± SD. **H** and **I**: **P < 0.01 and ***P < 0.001 by 1-way ANOVA with Tukey’s multiple comparisons test. All experiments were repeated 3 times.

To further elucidate the molecular mechanism of CD24 and RCC2 interaction in prostate cancer cells, we performed RNA sequencing (RNA-Seq) to identify differentially expressed genes (DEGs) in *RCC2*-KO and *CD24*-KO DU145 cells compared with scrambled control cells (Figure 7, A and B). Gene set enrichment analysis (GSEA) revealed that DEGs were enriched in the Wnt/β-catenin signaling pathway, which was downregulated in *CD24*-KO cells but upregulated in *RCC2*-KO cells compared with scrambled control cells (Figure 7, C and D). These results suggest differential regulation of the Wnt/β-catenin signaling pathway by RCC2 and CD24, indicating their potential roles in modulating this pathway in prostate cancer. Western blot analysis revealed that *RCC2* KO not only increased β-catenin activation but also decreased the expression of AXIN2 and APC, 2 inhibitors of β-catenin (Figure 7E). In contrast, *CD24* KO and *CD24/RCC2* KO led to β-catenin inactivation, suggesting that CD24 and RCC2 have opposing roles in the regulation of β-catenin signaling in prostate cancer cells. Given the increased RCC2 protein levels in *CD24*-KO DU145 cells and xenograft tumors, we further investigated whether *CD24*-KO–induced RCC2 expression is associated with the β-catenin signaling pathway. As shown in Figure 7F, RCC2 protein levels increased in both *CD24* KO and DU145 cells treated with XAV939, a β-catenin pathway inhibitor. Notably, β-catenin signaling activation was reduced following XAV939 treatment for 24 hours at a concentration of 50 μM. To further validate the role of CD24 in regulating RCC2 expression, we established a *CD24*-OE cell model in the CD24-negative prostate cancer cell line LNCaP by transducing the coding sequence of human *CD24* (7). Both the mRNA and protein levels of RCC2 were measured in this cell model. RCC2 protein levels decreased with increased β-catenin signaling activation in *CD24*-OE LNCaP cells but were restored in XAV939-treated *CD24*-OE LNCaP cells (Figure 7G). However, *CD24* overexpression led to a 2-fold increase in *RCC2* mRNA levels in LNCaP cells, but this effect was abolished in XAV939-treated *CD24*-OE LNCaP cells (Figure 7H), suggesting potential feedback in the transcriptional regulation of *RCC2*. AXIN2 is not only a negative regulator but also a transcriptional target of the β-catenin signaling pathway (35). As shown in Figure 7, G and I, increased mRNA and protein levels of AXIN2 were evident after *CD24* overexpression, but this increase was restored in XAV939-treated *CD24* OE LNCaP cells. Additionally, overexpression of vimentin was observed in *RCC2*-KO and *CD24/RCC2*-KO xenograft tumors, while vimentin expression was reduced in DU145 *CD24*-KO xenograft tumors compared with expression in scrambled control xenograft tumors (Figure 7J). In contrast, E-cadherin overexpression was evident in *CD24* KO xenograft tumors, and reduced vimentin expression was observed in *RCC2* KO and *CD24/RCC2* KO xenograft tumors (Figure 7J). Thus, DU145 cells exhibited a mesenchymal phenotype when *RCC2* was knocked out and an epithelial phenotype when *CD24* was knocked out, indicating the roles of RCC2 and CD24 in EMT. As *Vim* is a β-catenin target gene, CD24/RCC2-mediated EMT is likely to be regulated by the β-catenin signaling pathway. These data provided strong evidence that the interaction between CD24 and RCC2 modulates β-catenin signaling activation (Supplemental Figure 10), which is a key regulator of cell movement, adhesion, migration, invasion, and metastasis in prostate cancer cells.

## Discussion

CD24, previously recognized for its role in tumor growth through modulation of the ARF-NPM interaction and inactivation of the p53 pathway (34), has been shown to interact directly with RCC2, a guanine nucleotide exchange factor linked to cell migration and metastasis. In the present study, we identified a functional interaction between CD24 and RCC2 in prostate cancer and provided critical insights into the mechanisms underlying prostate cancer metastasis. The positive correlation and coexpression of CD24 and RCC2, along with CD24’s binding to both the C-terminal and N-terminal domains of RCC2, support a direct interaction between the 2 proteins, which may play a role in regulating cell motility and adhesion. Functional analysis revealed that *CD24* KO reduced cell proliferation, migration, and metastasis, whereas *RCC2* KO inhibited cell proliferation and enhanced the migratory capacity of prostate cancer cells. This dual behavior indicates that, while RCC2 plays a cooperative role with CD24 in promoting cell proliferation, it has an opposing effect on cell migration. In vivo experiments corroborated these findings, showing that *CD24* KO reduced tumor growth and metastasis, whereas *RCC2* KO primarily induced metastasis without altering tumor growth, underscoring the critical role of RCC2 in metastatic dissemination rather than primary tumor growth. Overall, these findings suggest a complex, context-dependent interplay between CD24 and RCC2 in prostate cancer.

This is the first evidence that RCC2 influences prostate cancer cell behavior through a distinct mechanism involving ubiquitination and degradation of vimentin, a key intermediate filament protein implicated in cell migration (36). We identified vimentin as a major RCC2 binding partner, and found that RCC2 directly interacted with and ubiquitinated vimentin, thereby targeting it for proteasomal degradation. These data underscore the role of RCC2 in regulating levels, and, consequently, cell migratory behavior. RCC2 and Vimentin have a negative correlation at both mRNA and protein levels in prostate cancer tissues, reinforcing the functional link between RCC2-mediated Vimentin degradation and reduced migration. Furthermore, *RCC2* KO resulted in compressed F-actin filaments and condensed microtubules, indicating an additional mechanism of RCC2-mediated reorganization of the cytoskeleton, which supports enhanced cell migration. While the α5/β1 FN-associated integrin network is essential for the constitutive invasiveness of cancer cells, *RCC2* KO did not alter the activation patterns of RAC1 and ARF6 in response to FN stimulation, suggesting that RCC2-regulated migration does not occur through the direct modulation of these GTPases. Collectively, these findings suggest that RCC2 plays a pivotal role in prostate cancer cell migration by regulating vimentin stability and cytoskeletal organization.

The present study highlights the molecular mechanism by which CD24 ubiquitinates and degrades RCC2, modulating β-catenin signaling, which is a critical driver of cancer progression and EMT (37). *CD24* KO leads to accumulation of RCC2 predominantly in the cytoplasm, which contrasts with RCC2 degradation upon CD24 overexpression. This degradation process is mediated by the ubiquitin-proteasome system, as evidenced by the stabilization of RCC2 following treatment with the proteasome inhibitor MG-132, and enhanced RCC2 ubiquitination in cells overexpressing CD24. In xenograft models, *CD24* KO tumors exhibited elevated RCC2 levels and an epithelial phenotype marked by increased E-cadherin and decreased vimentin expression, indicating EMT suppression. Conversely, *RCC2* KO induced a mesenchymal phenotype with increased vimentin expression, supporting the role of *RCC2* KO in the promotion of EMT and metastasis. Further elucidation of the functional interaction between CD24 and RCC2 revealed differential regulation of the Wnt/β-catenin signaling pathway, which was downregulated in *CD24*-KO cells but upregulated in *RCC2*-KO cells, suggesting opposing roles in this signaling cascade. Interestingly, the β-catenin pathway further modulated the transcriptional regulation of *RCC2*, indicating a feedback loop between RCC2 and β-catenin signaling. These findings suggest that CD24 and RCC2 cooperatively regulate the β-catenin signaling pathway, with CD24 acting as a suppressor of RCC2 through ubiquitination, thereby inhibiting β-catenin signaling and promoting an epithelial phenotype. This cooperative interaction underscores the complexity of the regulatory networks involving CD24 and RCC2 in prostate cancer.

Although it is well known that RCC2 plays an oncogenic role in tumor cell growth, its role in tumor progression and metastasis has been inconsistent across different studies, pathway interactions, and experimental conditions. RCC2 has diverse functions in various types of cancers. In lung cancer (20) and breast cancer (21), RCC2 promotes cell migration and metastasis through EMT, whereas RCC2 suppresses cell migration and metastasis via Rac1 inactivation in colorectal cancer (23). However, RCC2 acts as a p53 target and in p53-null colorectal cancer cells, ectopic expression of RCC2 restores directional cell migration (23). Notably, clinical data have shown that weak RCC2 protein expression is associated with poor prognosis in patients with colorectal cancer (38), supporting the tumor-suppressive role of RCC2. Furthermore, the effects of RCC2 are likely influenced by interactions with various pathways, such as the Rac1, Wnt, Hh/GLI1, and DNMT1 signaling pathways (15, 21, 23, 39, 40). Methodological differences, including variations in cell lines and experimental models, may further contribute to these discrepancies. The present study aligns with previous reports that RCC2 inhibits cell migration and metastasis in prostate cancer. However, a recent study (15) reported that, in prostate cancer cells, *RCC2* overexpression enhanced cell proliferation, migration, invasion, and EMT, whereas *RCC2* knockdown suppressed these processes. The present study is the first to develop various *RCC2*-KO prostate cancer cells and animal models and to establish the role of RCC2 in inhibiting cell migration, metastasis, and EMT in prostate cancer. These findings suggest a complex, context-dependent regulatory role of RCC2 in cancer, underscoring the need for further research to elucidate these discrepancies and mechanisms.

In summary, the present study underscores the nuanced roles of CD24 and RCC2 in prostate cancer, in which CD24 acts as a regulator of RCC2 stability and function, affecting the critical pathways that govern cell proliferation, migration, and EMT. This complex regulatory axis between the CD24, RCC2, Vimentin, and β-catenin pathways offers new insights into the molecular drivers of prostate cancer and highlights potential targets for therapeutic interventions aimed at disrupting the metastatic capabilities of prostate cancer cells.

## Methods

### Sex as a biological variable.

Our study exclusively examined male mice because prostate cancer is a sex-specific disease that only develops in male animals. Since the prostate is a male-specific organ, the biological phenotype of prostate cancer cannot be studied in female mice. Therefore, the inclusion of only male mice is scientifically justified based on the nature of the disease being investigated.

### Cell lines, plasmids, antibodies, and reagents.

Prostate cancer cell lines, including PC3 (Cat. CRL-1435), and PC3-luc cells (Cat No. CRL-1435) and DU145 cells (Cat. HTB-81) and LNCaP cells (Cat. CRL-1740) were obtained from American Type Culture Collection (ATCC). To maintain integrity, the cell lines were freshly expanded and cryopreserved shortly after acquisition from the ATCC, with renewal every 5 months. Authentication was confirmed via professional verification services, ensuring no contamination, and cell line identity was validated using short tandem-repeat (STR) DNA profiling. The culture conditions included Dulbecco’s modified Eagle’s medium (DMEM) for DU145 and PC3 cells and Roswell Park Memorial Institute (RPMI) 1640 medium for LNCaP cells, both supplemented with 10% phosphate-buffered saline (PBS) sourced from Thermo Fisher Scientific. The primary antibodies used in this study are listed in Supplemental Table 3. The PX458 plasmid was obtained from Addgene, while the p3XFLAG-CMV-7.1 plasmid was procured from Millipore Sigma. CD24 and RCC2 single-guide RNAs (sgRNAs) and primers were synthesized by Integrated DNA Technologies. Additionally, tamoxifen from Millipore-Sigma was used to induce Cre recombinase activity in the mouse models.

### Generation of knockout and overexpressed cell lines.

sgRNAs were designed using Benchling’s online CRISPR design platform (Benchling, https://benchling.com), which generates a ranked list based on specificity and efficiency scores. Paired sgRNAs with specificity and efficiency scores exceeding 30% were selected for the targeting sites flanking the target gene sequence. Oligonucleotides corresponding to the selected targeting sites were annealed and inserted into the pSpCas9(BB)-2A-GFP (pX458) vector, which was linearized using BbsI (Addgene). Cell transfection was performed using Lipofectamine 3000 with either the pX458 plasmid harboring the target sgRNA sequences or an empty pX458 vector. After transfection, green fluorescent protein–positive (GFP-positive) cells were sorted by flow cytometry, and 200 GFP^+^ cells were plated in 10-cm dishes for clonal expansion. KO clones were identified by Sanger sequencing of genomic DNA and target gene expression levels were verified by Western blot analysis. To generate control cells, scrambled sgRNA without a specific target was introduced into the cells using Cas9, and the resulting clones were confirmed by GFP sorting, followed by Sanger sequencing. Off-target analysis of all sgRNAs was performed using Cas-OFFinder (http://www.rgenome.net/cas-offinder), and potential off-target effects were excluded by PCR and sequencing of the off-target regions. Detailed sequences of sgRNAs and primers used are provided in Supplemental Table 4. For overexpression studies, the pLVX-Puro-CD24-GFP vector was transfected into LNCaP cells to enhance CD24 expression, which was subsequently validated using Western blotting.

### Cell growth assay and colony formation assay.

For the cell growth assay, the cells were initially cultured in 6-well plates in DMEM supplemented with 10% FBS until they reached approximately 70% confluency. Following 2 PBS washes, the cells were detached, centrifuged, and seeded into 24-well plates at a density of 5,000 cells per well for growth assessment and into 6-well plates at 400 cells per well for the colony formation assay. Cell proliferation was monitored by manually counting the cells under a microscope on days 1, 2, 3, 4, and 5, and cell growth curves were plotted based on these counts. Viable cells were identified using the dye exclusion method. For the colony formation assay, cells were cultured for 2 weeks, washed 3 times with PBS, and fixed with 4% paraformaldehyde (PFA) for 20 minutes. Following fixation, cells were again washed thrice with PBS and stained with 0.1 g/mL crystal violet for 20 minutes. The plates were then scanned to assess colony size and number, with colonies defined as groups of 50 or more cells under microscopic evaluation. For the soft agar colony formation assay, cells were seeded at 10^5^ cells per well on a solidified layer of culture medium in multiwell plates, with the medium replaced every 3 days. After 3–4 weeks of incubation, colonies were washed with PBS and imaged using a digital camera (Thermo Fisher Scientific). The 3D colonies were counted based on their ability to form clusters of at least 50 cells from a single cell (34).

### Transwell migration assays and automated scratch migration assays.

The cells were cultured in DMEM supplemented with 10% FBS. For the Transwell migration assay, cells were resuspended in DMEM containing 0.2% FBS and seeded into 8-μm pore invasion chambers (Millipore Sigma) at a density of 5 × 10^4^ cells per well. After a 20-hour incubation period, the chambers were rinsed 3 times, and the nonmigrating cells on the upper side were removed by scraping. The migrated cells on the underside were fixed with 4% paraformaldehyde (PFA), washed with PBS, and stained with either 4′,6-diamidino-2-phenylindole (DAPI) or hematoxylin for 10 minutes. After drying in the dark, cells were imaged under a microscope.

### IF.

For immunofluorescence, 10^4^ cells were plated onto 8-well chamber slides and allowed to adhere for 20 hours. The cells were fixed with 4% PFA and permeabilized with 0.25% Triton X-100 for 10 minutes. Following permeabilization, cells were blocked with PBS containing 2% goat serum for 1 hour. The primary antibody was then added and the cells were incubated overnight at 4°C. Subsequently, secondary antibodies were applied for 1 hour, and the nuclei were counterstained with DAPI for 10 min. After thorough washing, coverslips were mounted onto glass slides for visualization.

### Quantitative image analysis of protein colocalization.

To quantify the colocalization of 2 proteins in IF images, we used the JaCoP plugin in ImageJ/Fiji (https://imagej.net/plugins/jacop) (41) to calculate 2 standard colocalization metrics: Pearson’s correlation coefficient (PCC) and Manders’ overlap coefficient (MOC). PCC evaluates the linear relationship between the fluorescence intensities of the 2 proteins, with values ranging from –1 (perfect anti-correlation) to +1 (perfect correlation), and 0 indicating no correlation. MOC measures the proportion of one protein’s fluorescence that overlaps with the other, providing a precise assessment of colocalization. A value close to 1 indicates a high degree of overlap, while a value near 0 indicates minimal or no colocalization. By analyzing these coefficients, we quantitatively assessed the degree of colocalization between the 2 proteins, thereby gaining insights into their spatial association in the imaged samples.

### Western blots and immunoprecipitation.

Western blotting was conducted as previously described (34), and the proteins were visualized using the ChemiDoc MP Imaging System (Bio-Rad) or Mini Med 90 Processor (AFP). For IP assays, cells were lysed in cold buffer containing protease inhibitors and phenylmethylsulfonyl fluoride (PMSF) (Millipore Sigma) for 15 minutes. Extracted proteins were divided into separate tubes and incubated with IgG and a specific primary antibody at room temperature for 24 hours. The antibody-protein complexes were precipitated using protein A/G agarose beads (Thermo Fisher Scientific).

### High-resolution mass spectrometry analysis.

For sample processing, the gel bands or spots were excised into 1 mm³ cubes and initially washed with water, followed by 3 washes with 25 mM ammonium bicarbonate in 50% acetonitrile. The gel pieces were dehydrated using acetonitrile and disulfide bonds were reduced by incubating the samples with 10 mM DTT in 25 mM ammonium bicarbonate buffer at 56°C for 60 minutes. Cysteine alkylation was achieved by incubating samples with 55 mM iodoacetamide in the same buffer for 45 minutes in the dark at room temperature. After dehydration, the gel pieces were covered with a trypsin solution (10 ng/μL in 25 mM ammonium bicarbonate buffer) and incubated on ice for 30 minutes. The remaining trypsin solution was removed and 25 mM ammonium bicarbonate was added for overnight proteolysis at 37°C. Proteolysis was terminated by adjusting the concentration of the sample to 5% formic acid. Peptides were extracted from the gel by sequential washing with 0.1% formic acid in 50% and 100% acetonitrile. The extracts were combined and vacuum dried. Peptide separation was performed by resuspending the samples in buffer A (2% acetonitrile and 0.1% formic acid) and centrifuging them. Peptides were loaded onto a Shimadzu LC-20AD nanoHPLC system and eluted onto an in-house packed C18 analytical column with a gradient of buffer B (98% acetonitrile and 0.1% formic acid) coupled with a mass spectrometer for further analysis.

For analysis, peptide samples were ionized using nanoelectrospray ionization and analyzed by tandem mass spectrometry (MS/MS) using a Q Exactive mass spectrometer. Data-dependent acquisition (DDA) mode was employed with an initial resolution of 70,000 for intact peptides. Peptides were selected for MS/MS using high-energy collision dissociation (HCD) at a normalized collision energy setting of 27. The resulting ion fragments were detected at a resolution of 17,500 using an Orbitrap. A DDA procedure was followed, alternating between one MS scan and 15 MS/MS scans, targeting the most abundant precursor ions exceeding a threshold of 20,000 counts, with a dynamic exclusion duration of 15 seconds. The mass range of the MS scans was 350–2000 Da. Data analysis was performed using Proteome Discoverer software (ver. 1.3.0.339) to convert raw data files to.mgf format, and the Mascot search engine (ver. 2.3.0) was used for peptide and protein identification. Searches were restricted to tryptic peptides, with carbamidomethylation (C) as a fixed modification and oxidation (M) and Gln→pyro-Glu (N-term Q) as variable modifications. One missed cleavage was allowed and the precursor error tolerance was set to 10 ppm, with a fragment deviation of 0.1 Da. The identified peptides were grouped into proteins and the results were stored for further quantitative analysis.

### Transplantation of xenogeneic tumor cells.

NOD-scid IL2rgnull (NSG) immunodeficient mice were obtained from Jackson Laboratory. Scrambled or knockout (KO) cells (10^6^ cells in 100 μL) were subcutaneously injected into the left flank of 8-week-old male NSG mice. The growth and metastasis of xenograft tumors were monitored using firefly luciferase bioluminescence imaging at 10-day intervals for up to 60 days. The tumor size and weight were measured as previously described (34). The mice were euthanized for histopathological assessment and additional analyses. In another experiment, scrambled and KO cells were combined (100 μL, 10^6^ cells) and injected into the dorsal abdomen of male NSG mice aged 6–8 weeks. Tumors and lung tissues were collected for histological examination and expression analysis, and the number of tumor nodules across all the lung lobes was scored in a blinded manner.

### Genetically engineered animal models.

To generate genetically engineered mouse models, *Rcc2* and *Pten* floxed mice (The Jackson Laboratory) were crossed with *Nkx3-1*^CreERT2^ knock-in mice (National Cancer Institute Mouse Model Deposit) that express Cre recombinase under tamoxifen-inducible control on a C57BL/6 background. This breeding strategy produced conditional knockout (cKO) male mice for prostate-specific deletion of *Rcc2*, *Pten*, or both genes (*Nkx3-1*^CreERT2/–^ × *Rcc2*^fl/fl^, *Nkx3-1*^CreERT2/–^ × *Pten*^fl/fl^, and *Nkx3-1*^CreERT2/–^ × *Rcc2*^fl/fl^ × *Pten*^fl/fl^, respectively). Additionally, *Nkx3-1*^CreERT2/–^ × *Rcc2*^fl/fl^ mice were crossed with TRAMP mice (Jackson Laboratory) on a C57BL/6 background to generate prostate-specific *Rcc2* cKO TRAMP male mice (*Nkx3-1*^CreERT2/–^ × *Rcc2*^fl/fl^ × TRAMP). These mice were monitored for the development and metastasis of spontaneous prostate tumors for up to 12 months. Histological examination and expression analyses were performed as previously described.

### Prostate cancer specimens.

In the present study, 78 formalin-fixed, paraffin-embedded primary prostate cancer tissue specimens were used for IHC staining. The specimens collected from patients who underwent primary surgery between January 2012 and June 2018 at the University of Alabama at Birmingham included clinical data such as patient age, prostate-specific antigen (PSA) levels, Gleason score, and pathological stage (Supplemental Table 1). Prostate cancer diagnoses were confirmed by histopathological examination, with staging conducted according to the tumor-node-metastasis (TNM) classification system. Pathological grading was categorized based on the Gleason scores: 2–6, 7, and 8–10. The study protocol was approved by the Institutional Review Board (IRB) of the University of Alabama at Birmingham before commencement.

### IHC)analysis.

IHC staining was conducted using Vectastain Elite ABC kits (Vector Lab) according to established protocols (34). The extent of positive staining in tumor cells (ranging from 0% to 100% per tissue section) was multiplied by the staining intensity (1, weak; 2, mild; 3, strong) to generate H-scores ranging from 0 to 300.

### Quantitative polymerase chain reaction.

Total RNA was extracted from cultured cells using TRIzol reagent (Thermo Fisher Scientific), following the manufacturer’s instructions. For real-time PCR, 2 μL of the synthesized cDNA served as the template, and reactions were performed using a LightCycler 480 Real-Time PCR System (Roche Applied Sciences) with the miScript SYBR Green PCR kit (QIAGEN). The PCR conditions included an initial incubation at 95°C for 10 minutes, followed by 40 cycles of 95°C for 15 seconds and 60°C for 1 minute. The cycle threshold (Ct) values were determined under fixed threshold settings, with the mean Ct values calculated from triplicate reactions. The relative expression of the target genes was quantified using the 2^–ΔCt^ method and normalized to GAPDH as a reference gene. The 2^–ΔCt^ method allows the comparison of target gene expression relative to the reference gene. The primer sequences used for qPCR are listed in Supplemental Table 4.

### RNA-seq and bioinformatic analysis.

RNA libraries were prepared using the TruSeq Stranded mRNA Library Prep Kit (Illumina), according to the manufacturer’s protocol. RNA integrity was assessed using an Agilent 2200 Tapestation System. First-strand cDNA synthesis was performed using random hexamers and ProtoScript II Reverse Transcriptase (New England Biolabs, UK). The prepared libraries were then normalized, pooled, and sequenced on an Illumina HiSeqX10 system with paired-end reads for 150 cycles in accordance with the manufacturer’s instructions. Differential expression analysis of genes (DEGs) was conducted by evaluating fold changes and q-values. Functional grouping and visualization of terms and pathways for extensive gene clusters were performed using the ClueGO plug-in on the Cytoscape platform (apps.cytoscape.org/apps/cluego). Gene clusters were either imported from text files or extracted interactively from the Cytoscape network. For further insight, KEGG pathway analysis (www.genome.jp/kegg/) was conducted on the identified DEGs to explore the interacting genes and proteins using the STRING database (string-db.org/). Interaction networks of the DEGs were constructed and visualized using the Cytoscape software (Cytoscape.org/).

### Correlation analysis between CD24 and other transcripts.

A total of 554 transcriptomic profiles from the TCGA-PRAD cohort were obtained. Following quality control and data cleaning, duplicate samples from the same patient were removed, retaining only the most recent follow-up data for each patient. As a result, 424 unique samples were included in the final analysis. Correlation coefficients between CD24 and all other gene transcripts were calculated using Pearson correlation analysis. A volcano plot was generated, with the Pearson correlation coefficient (*R*) on the *x*-axis and the adjusted *P*-value (FDR) on the *y*-axis. Genes with a correlation coefficient *r* > 0.30 or *r* < −0.30, and an FDR < 0.05, were considered statistically significant and selected as meaningful candidates for further analysis.

### RCC2 correlation with metastasis-related pathways.

A total of 554 transcriptomic profiles from the TCGA-PRAD dataset were initially obtained. After sample screening and data cleaning, 550 high-quality samples were retained for downstream analysis. Genomic variation and pathway activity scores were estimated using the R package GSVA (Gene Set Variation Analysis), with the complete MSigDB gene set used as the reference database. From the resulting GSVA-derived pathway matrix, pathways associated with metastasis were selected for further investigation. Pearson’s product-moment correlation was applied to assess the relationship between *RCC2* expression levels and pathway activity scores across the samples. Multiple testing correction was performed using the Benjamini-Hochberg or Bonferroni method. Only pathways showing a statistically significant correlation with *RCC2* expression (FDR < 0.05, 2-tailed test) were retained for further analysis, allowing for the evaluation of *RCC2*’s association with metastasis-related and other biologically relevant pathways in prostate cancer.

### Statistics.

Statistical analyses were conducted using the Mann–Whitney U test or 2-tailed *t* test for comparisons between 2 groups. For comparisons involving more than 2 groups, 1-way ANOVA was applied, followed by post hoc assessments. Two-way repeated-measures ANOVA was used to evaluate differences over time among the groups. Kaplan-Meier survival curves, as well as the initiation and metastasis of tumors and survival outcomes in mice, were analyzed using the log-rank test. All statistical analyses were performed using 2-tailed tests. Reproducibility was assessed by repeating the in vitro experiments 3 times and the in vivo experiments twice, each yielding consistent results. Data analysis was performed using Microsoft Excel (Microsoft 365) and GraphPad Prism (version 10).

### Study approval.

All animal studies were conducted in the Animal Resources Program at the University of Alabama at Birmingham, with environmental conditions maintained at 18–24°C, 40%–60% humidity, and a 12-hour light/dark cycle. The facility also provides onsite veterinary care. All experimental procedures were approved by the Institutional Animal Care and Use Committee (IACUC) of the University of Alabama at Birmingham, following established guidelines for the care and use of laboratory animals.

### Data availability.

Values for all data points in graphs are reported in the Supporting Data Values file. Genetic alterations and gene expression data were sourced and annotated from publicly accessible datasets, including the cBioPortal for Cancer Genomics, Prostate Cancer Transcriptome Atlas (PCTA), and Cancer Genome Atlas (TCGA) data portal. Data specific to prostate adenocarcinomas were analyzed for genetic alterations using the cBioPortal (www.cbioportal.org). RNA expression analysis from TCGA and PCTA datasets was conducted using cBioPortal, PCTA (www.thepcta.org), and UALCAN (ualcan.path.uab.edu). Survival analyses based on gene expression from TCGA dataset were performed using GEPIA (gepia.cancer-pku.cn). The RNA-seq data generated in this study were deposited in the NCBI GEO database under accession no. GSE277962. Additionally, mass spectrometry proteomics data were submitted to the ProteomeXchange Consortium via the PRIDE partner repository (www.ebi.ac.uk/pride/) under the dataset identifier PXD056404.

## Author contributions

Conceptualization: RL, LW, and JZ. Methodology: XC, YW, HY, and ZL. Data analysis: XC, YW, HY, ZL, CZ, LW, and RL. Investigation: XC, YW, HY, ZL, and CZ. Resources: JZ, LW, and RL. Writing: RL, JZ, LW, and XC. Visualization: XC, HY, and ZL. Supervision: RL, JZ, and LW. Funding acquisition: RL and JZ. The order of authorship was determined based on their overall contributions to the project.

## Supplementary Material

Supplemental data

Unedited blot and gel images

Supporting data values

## Version 1 10/15/2025

Electronic publication
