# Supplementary material for: RCC2 and CD24 cooperate to modulate prostate cancer progression through vimentin ubiquitination and β-catenin activation
Source: J Clin Invest. 2025 Oct 15;135(20):e192883. doi: 10.1172/JCI192883 (PMC12520681; doi:10.1172/JCI192883)
Supplement: Supplemental data [file jci-135-192883-s278.pdf]

Supplementary Figures and Tables

RCC2 and CD24 Cooperate to Modulate Prostate Cancer Progression Through Vimentin Ubiquitination and β-Catenin Pathway Activation

Xuelian Cui[1], Yicun Wang[1], Chao Zhang[1], Haiyan Yu[1], Zhichao Liu[1], Lizhong Wang[1,2], Jiangbing Zhou[3,4], and Runhua Liu[1,2]

Authors' Affiliations: [1] Department of Genetics and [2]O'Neal Comprehensive Cancer Center, University of Alabama at Birmingham, Birmingham, AL. Department of [3]Neurosurgery and [4]Biomedical Engineering, Yale University, New Haven, CT.

Fig. S1

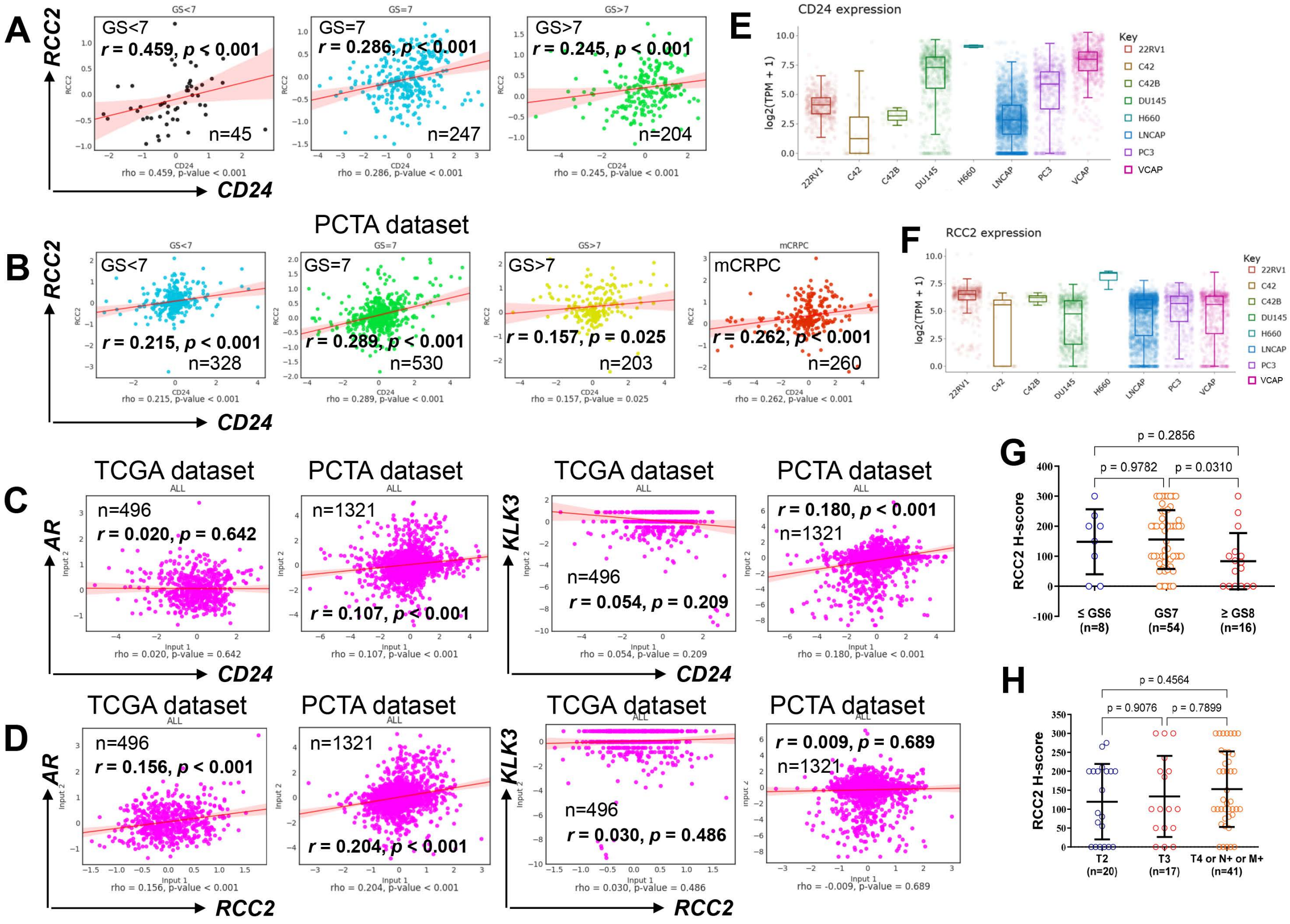

Supplementary Figure 1. Correlation between *CD24* and *RCC2* expressions in prostate cancer tissues and their association with AR signaling and tumor progression. (**A-D**) Scatter plots showing mRNA expression levels of *CD24*, *RCC2*, *AR*, and *KLK3* in human prostate cancer tissues from The Cancer Genome Atlas Program (TCGA) dataset and the Prostate Cancer Transcriptome Atlas (PCTA) dataset. (**E, F**) *CD24* and *RCC2* expressions across different human prostate cancer cell lines. This box- and -whisker plot with overlaid scatter points displays the log2 (TPM+1) values of mRNA expression. (**G, H**) Immunohistochemical (IHC) analysis of protein expression levels of CD24 and RCC2 with Gleason score and TNM stages in 78 primary prostate cancer samples. GS, Gleason score; TNM stages, T2, T3, T4, N+, or M+. **A**, **B**, **C**, and **D**: *r* was determined using Pearson's correlation test. **E** and **F**: Data are presented as medians and interquartile ranges. **G** and **H**: Data are presented as means ± SD, and *p* value was determined by one-way ANOVA with Tukey's multiple comparisons test.

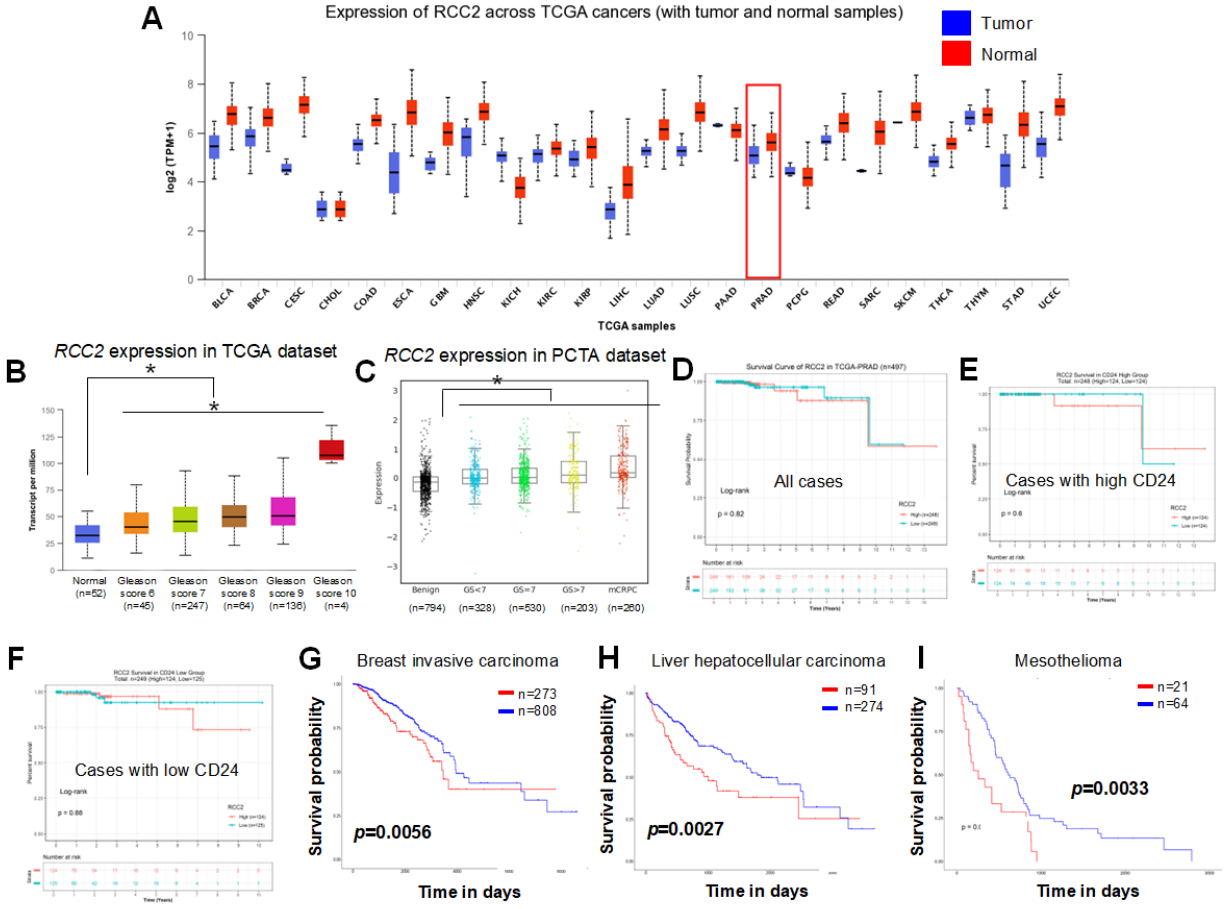

Supplementary Figure 2. *RCC2* expression and clinical relevance in human prostate cancer and other cancers. (**A**) Bioinformatics analysis of the TCGA dataset showing *RCC2* expression across 24 human cancer types, including prostate cancer. (**B**) *RCC2* expression in prostate adenocarcinoma samples with various Gleason scores compared to normal prostate samples (TCGA dataset). (**C**) *RCC2* expression in prostate cancer samples associated with Gleason score, tumor stage, and metastasis (PCTA dataset). (**D-F**) Overall patient survival analysis in prostate cancer patients stratified by *RCC2* and *CD24* expression levels (TCGA dataset). (**G-I**) Kaplan-Meier survival curves of *RCC2* expression in patients with breast invasive carcinoma, liver hepatocellular carcinoma, and mesothelioma based on TCGA data analysis. **A**, **B**, and **C**: Data are presented as medians and interquartile ranges, as determined by Kruskal–Wallis or Mann–Whitney U tests. *$p < 0.05$. **D**, **E**, **F**, **G**, **H**, and **I**: The log-rank test was used for patient survival analysis.

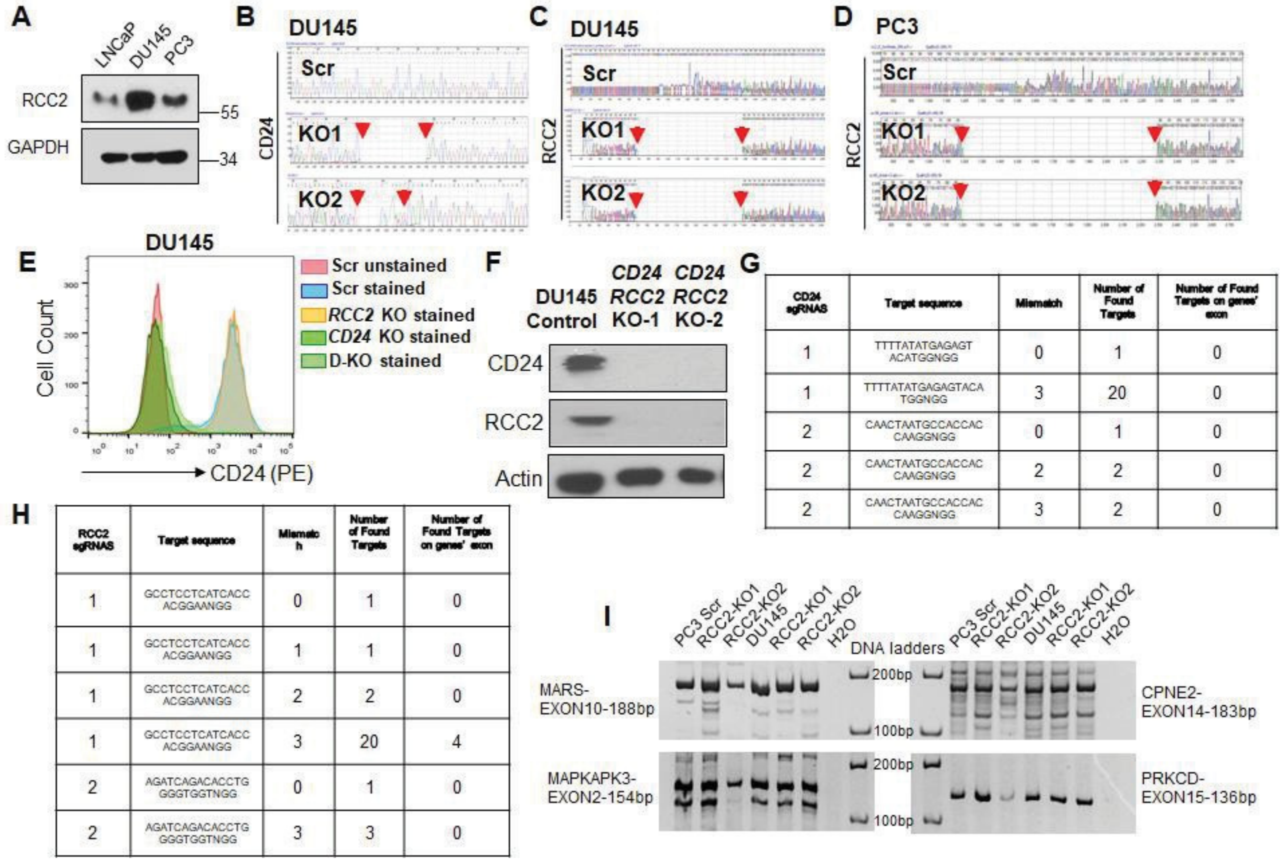

CD24 sgRNAS	Target sequence	Mismatch	Number of Found Targets	Number of Found Targets on genes' exon
1	TTTTATATGAGAGTACATGGNGG	0	1	0
1	TTTTATATGAGAGTACATGGNGG	3	20	0
2	CAACTAATGCCACCACCAAGGNGG	0	1	0
2	CAACTAATGCCACCACCAAGGNGG	2	2	0
2	CAACTAATGCCACCACCAAGGNGG	3	2	0

RCC2 sgRNAS	Target sequence	Mismatch	Number of Found Targets	Number of Found Targets on genes' exon
1	GCCTCCTCATCACCACGGAANGG	0	1	0
1	GCCTCCTCATCACCACGGAANGG	1	1	0
1	GCCTCCTCATCACCACGGAANGG	2	2	0
1	GCCTCCTCATCACCACGGAANGG	3	20	4
2	AGATCAGACACCTGGGGTGGTNGG	0	1	0
2	AGATCAGACACCTGGGGTGGTNGG	3	3	0

Supplementary Figure 3. Establishment and validation of *CD24* and *RCC2* knockout prostate cancer cell models. (**A**) Western blot analysis showing RCC2 expression levels in three human prostate cancer cell lines. (**B, C**) Establishment of *CD24* knockout (KO) DU145 cell lines (two clones) and *RCC2* KO DU145 cell lines (two clones) using CRISPR/Cas9 genome editing with two distinct single guide RNAs (sgRNAs). Validation of these cell lines was performed through Sanger sequencing. (**D**) Establishment of *RCC2* KO PC3 cell lines (two clones), validated by Sanger DNA sequencing. (**E**) Flow cytometry analysis showing CD24 expression in scramble (Scr), *CD24* KO, *RCC2* KO, and *CD24/RCC2* double KO (D-KO) DU145 cells. (**F**) CD24 and RCC2 expressions in *CD24/RCC2* double KO DU145 cells. (**G, H**) Cas-OFFinder analysis predicting potential off-target regions of *CD24* and *RCC2* sgRNAs, identifying four nucleotide mismatched genes: MARS, MAPKAPK3, CPNE2, and PRKCD for *RCC2* sgRNAs. (**I**) Denaturing gradient gel electrophoresis confirming no off-target effects on the predicted genes in the *RCC2* KO cell models.

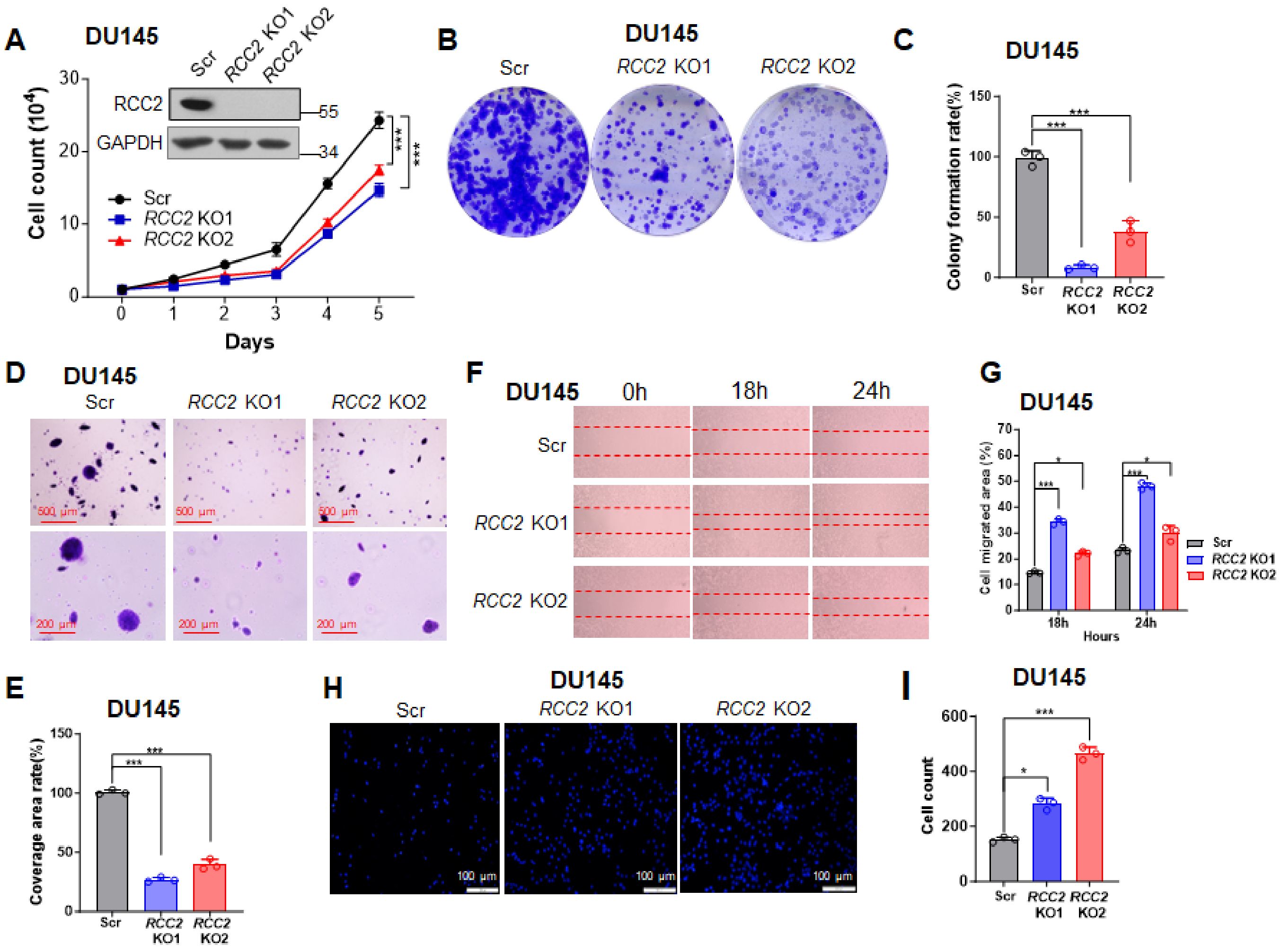

Supplementary Figure 4. The effect of *RCC2* knockout on cell proliferation and migration in DU145 cells. *RCC2* knockout (KO) DU145 cells exhibit reduced cell proliferation compared to scrambled control cells, as assessed by cell growth assay (**A**), colony formation (**B, C**), and soft agar assays (**D, E**). A representative Western blot showing RCC2 expression in *RCC2* KO DU145 cells is included as an inset in (**A**). *RCC2* KO also affects cell migration in DU145 cells compared to scrambled controls, as demonstrated by wound healing (**F, G**) and Transwell migration assays (**H, I**). Representative images and quantification of the assays are shown. Scale bars are indicated in the respective panels. Data are presented as the mean ± SD of triplicate samples. Statistical significance was determined using ANOVA with Tukey's post-hoc tests (**C**, **E**, **G**, and **I**) or two-way ANOVA (**A**), where *p < 0.05, **p < 0.01, and ***p < 0.001 compared to scrambled control cells.

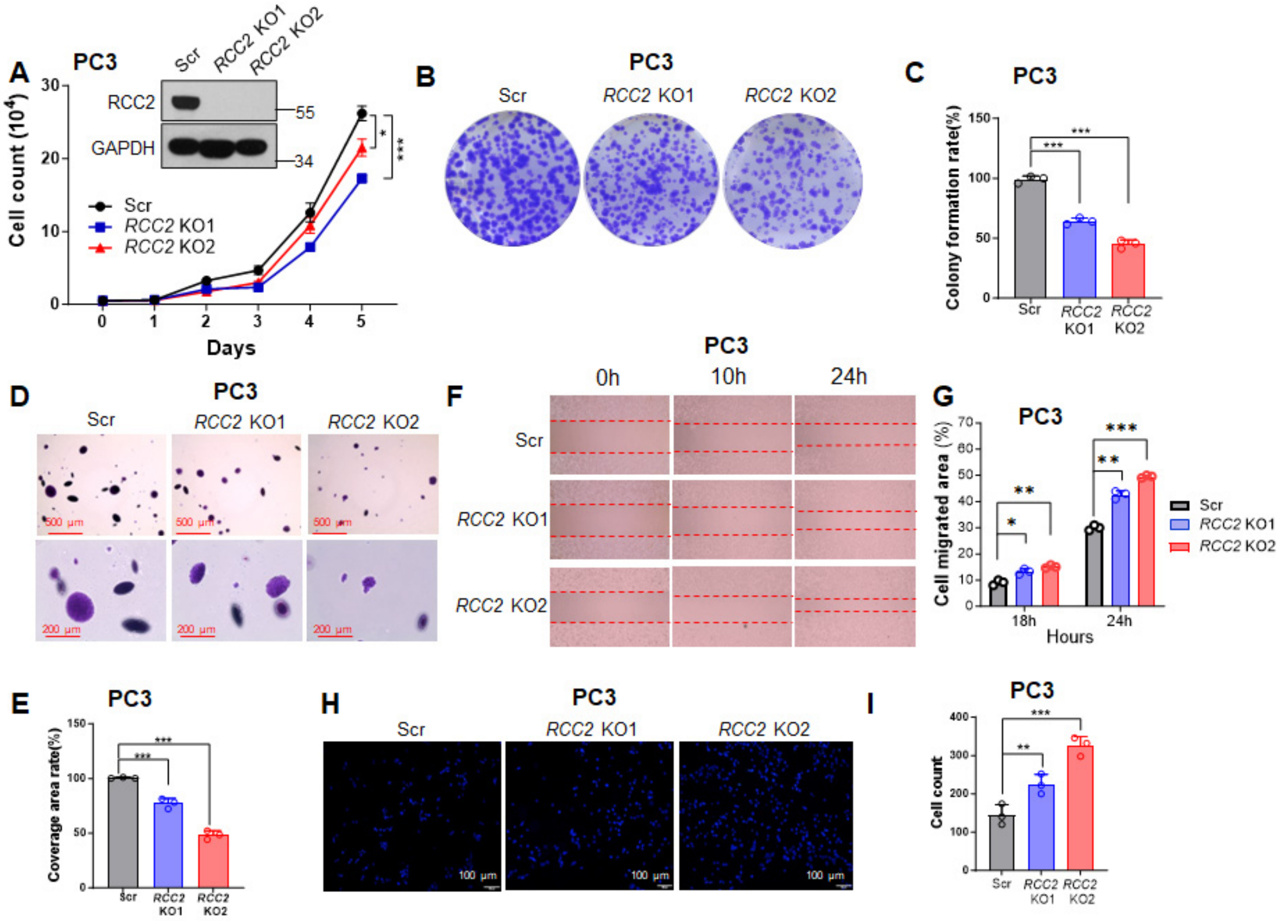

Supplementary Figure 5. The effect of *RCC2* knockout on cell proliferation and migration in PC3 cells. *RCC2* knockout (KO) PC3 cells exhibit reduced cell proliferation compared to scrambled control cells, as assessed by cell growth assay (**A**), colony formation (**B, C**), and soft agar assays (**D, E**). A representative Western blot showing RCC2 expression in *RCC2* KO PC3 cells is included as an inset in (**A**). *RCC2* KO also affects cell migration in PC3 cells compared to scrambled controls, as demonstrated by wound healing (**F, G**) and Transwell migration assays (**H, I**). Representative images and quantification of the assays are shown. Scale bars are indicated in the respective panels. Data are presented as the mean ± SD of triplicate samples. Statistical significance was determined using ANOVA with Tukey's post-hoc tests (**C**, **E**, **G**, and **I**) or two-way ANOVA(**A**), where $*p < 0.05$, $**p < 0.01$, and $***p < 0.001$ compared to scrambled control cells.

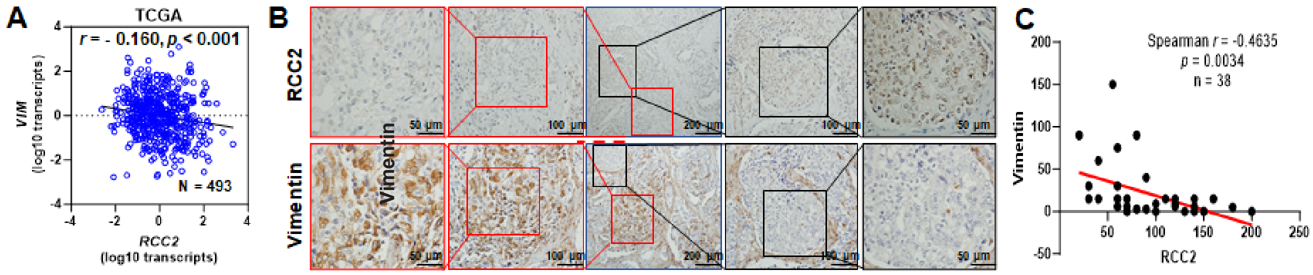

Supplementary Figure 6. Correlation of RCC2 with Vimentin expression in human prostate cancer tissues. (**A**) Scatter plots showing correlations in mRNA expression levels between *RCC2* and *VIM* in the TCGA dataset of primary prostate cancer tissues. r, correlation coefficient, determined by Pearson's correlation test. (**B**) Immunohistochemical (IHC) analysis of Vimentin protein expression in 38 primary prostate adenocarcinoma samples. Representative IHC images of Vimentin staining are shown. Scale bars are indicated in the respective panels. (**C**) H-score quantitative analysis showing correlations in protein expression levels between RCC2 and Vimentin. **A** and **C**: *r*, correlation coefficient, determined by Spearman's correlation test.

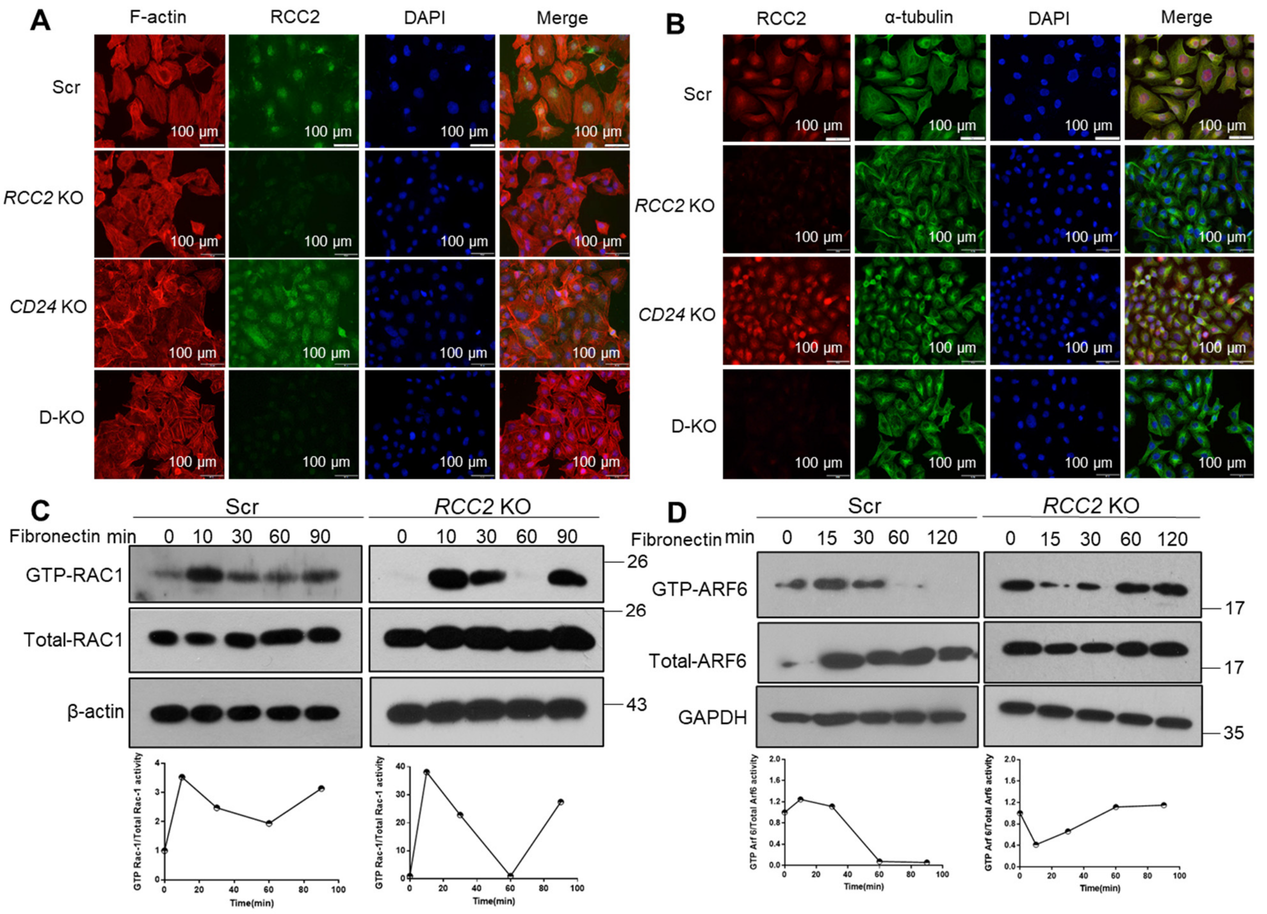

Supplementary Figure 7. Effect of *CD24* and *RCC2* knockout on cytoskeletal organization and GTPase signaling pathways in DU145 cells. (**A**) Immunofluorescence (IF) analysis of F-actin organization. (**B**) IF analysis of α-tubulin microtubule organization in scrambled control (Scr), *CD24* knockout (KO), *RCC2* KO, and *CD24/RCC2* double KO (D-KO) DU145 cells. Scale bars are indicated in the respective panels. Time-course analysis of (**C**) GTP-RAC1 expression and (**D**) GTP-ARF6 expression in scrambled control and *RCC2* KO cells following fibronectin (FN) stimulation.

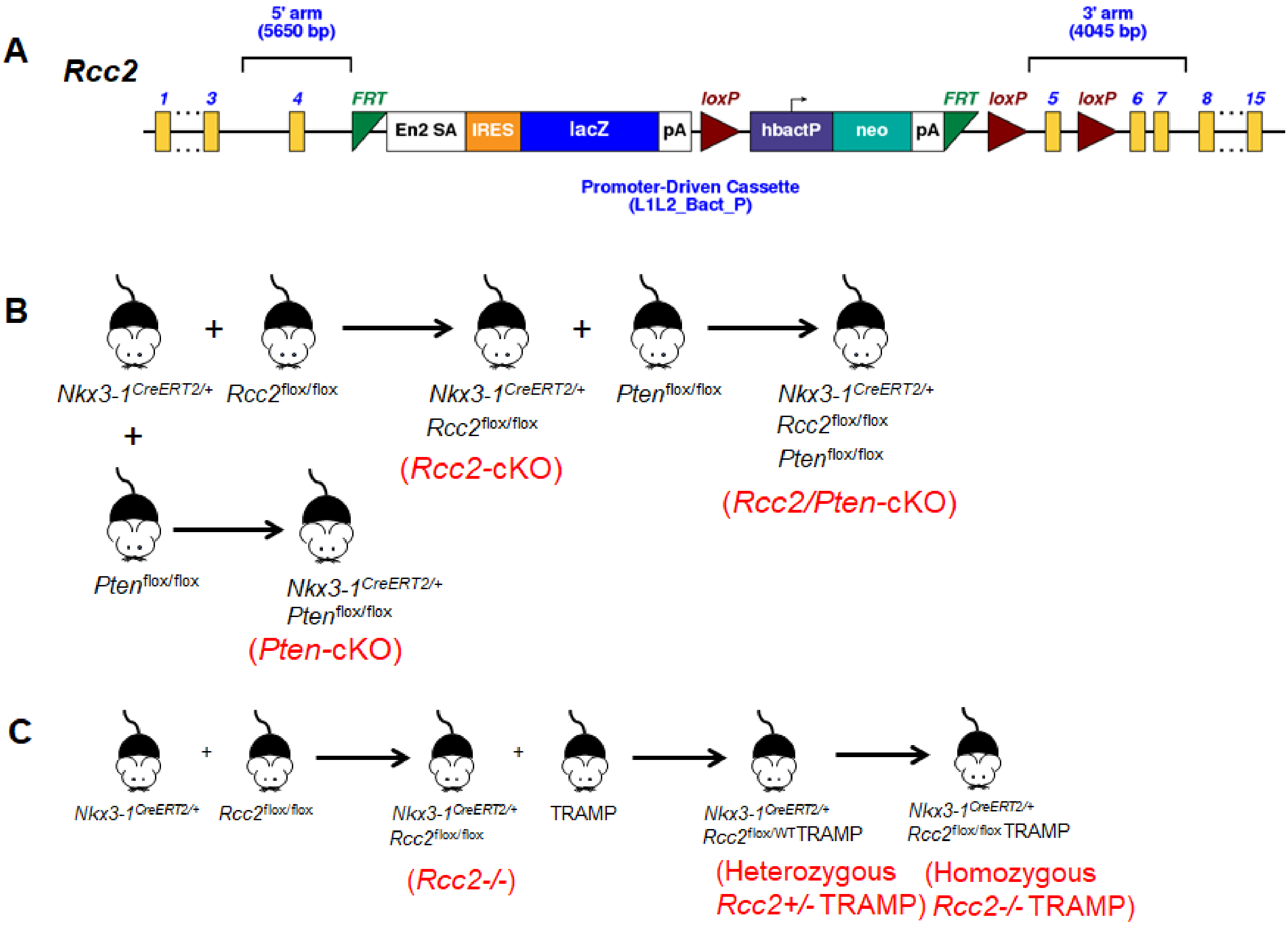

Supplementary Figure 8. Generation and validation of *Rcc2* and *Pten* conditional knockout mouse models. (**A**) Diagram illustrating the targeting construct used to generate the *Rcc2* floxed allele in mice. The construct includes a 5' arm (5,650 bp) and a 3' arm (4,045 bp) flanking the exon regions of the *Rcc2* gene. It features an FRT-flanked promoter-driven neomycin resistance gene (neo) for positive selection, an IRES-lacZ reporter for monitoring expression, and loxP sites flanking exons to allow for Cre- mediated recombination. The En2 splice acceptor (SA) is included upstream of the IRES- lacZ sequence to facilitate correct splicing. Two loxP sites are positioned to excise critical exons upon Cre recombinase activation, enabling conditional knockout of *Rcc2* in targeted tissues. The FRT sites allow for subsequent removal of the neomycin selection cassette via Flp recombinase. (**B**) Schematic representation of breeding strategies used to generate prostate-specific conditional knockout (cKO) models on a C57BL/6 background: *Rcc2*-cKO, *Pten*-cKO, and *Rcc2/Pten*-cKO. *Rcc2*-cKO mice were generated by crossing *Nkx3-1*^{CreERT2} knock-in mice with *Rcc2* floxed mice. *Pten*-cKO mice were generated by crossing *Nkx3-1*^{CreERT2} knock-in mice with *Pten* floxed mice, and *Rcc2/Pten*- cKO mice were generated by crossing *Nkx3-1*^{CreERT2} knock-in mice with both *Rcc2* and *Pten* floxed mice. (**C**) Schematic of crossing *Rcc2* cKO alleles into the transgenic adenocarcinoma of the mouse prostate (TRAMP) model on a C57BL/6 background to evaluate the role of *Rcc2* in spontaneous tumor metastasis. The models include homozygous *Rcc2*-cKO TRAMP and heterozygous *Rcc2*-cKO TRAMP mice.

Fig. S9

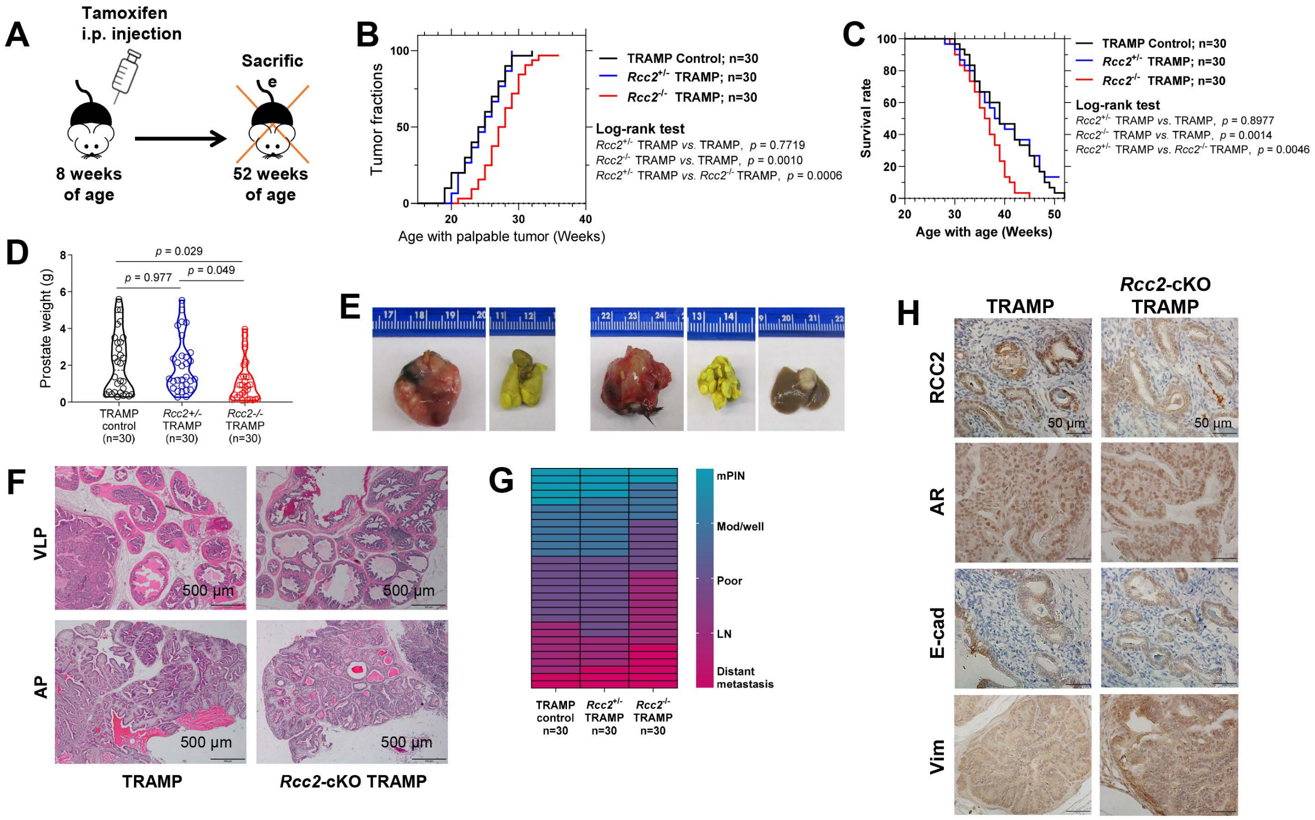

Supplementary Figure 9. Prostate-specific deletions of *Rcc2* and prostate tumor progression and metastasis in TRAMP models. (**A**) Schematic diagram of spontaneously developed prostate tumors followed up to 52 weeks of age in genetically engineered mouse models. Tamoxifen was administered at 8 weeks of age to induce Cre-mediated recombination in prostate epithelial cells. (**B, C**) Kaplan–Meier curves of palpable prostate tumors and survival, followed up to 36 and 52 weeks of age, respectively. (**D**) Prostate weights at 30 weeks of age. (**E, F**) Representative images of prostate tumor growth, metastasis, and H&E staining at 52 weeks of age. (**G**) Heatmap showing prostate tumor progression and metastasis at 30 weeks of age. (**H**) Representative immunostaining for RCC2, AR, E-cadherin, and Vimentin in prostates of mice at 30 weeks of age. Scale bars are indicated in the respective panels. **B** and **C**: The log-rank test was used to analyze mouse survival and tumor development to compare the distribution of time to event between groups. **D**: *p*-values were determined by one-way ANOVA with Tukey's multiple comparisons test. AR, androgen receptor; AP, anterior prostate; cKO, conditional knockout; LN, lymph node; Mod/well, moderately/well-differentiated grades; i.p., intraperitoneal injection; VP, ventral prostate. All experiments were repeated twice.

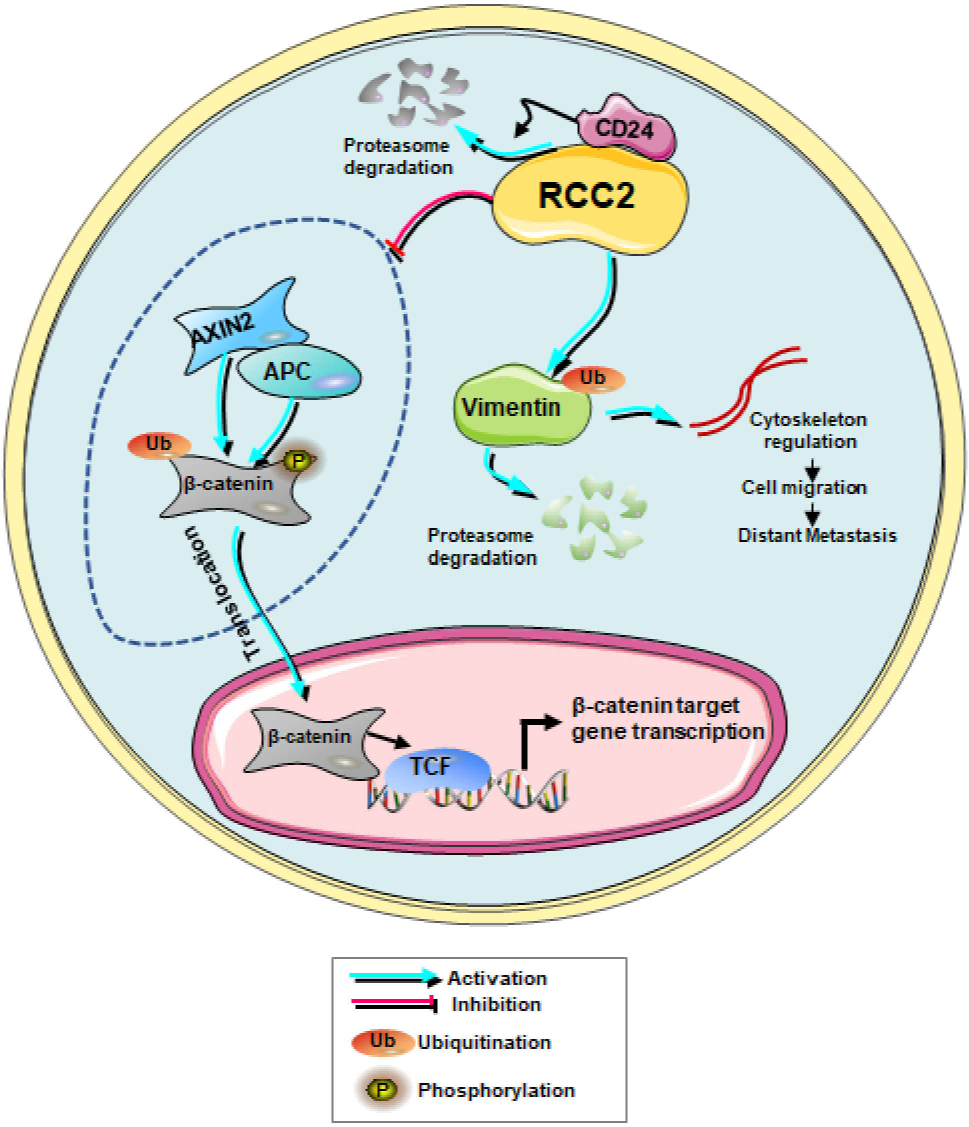

Supplementary Figure 10. Schematic representation of the proposed mechanism involving RCC2 and CD24 in regulating Wnt/β-catenin signaling and cell migration in prostate cancer. This figure illustrates the interaction between RCC2, CD24, and key components of the Wnt/β-catenin signaling pathway in prostate cancer cells. RCC2 regulates β-catenin stabilization and translocation, promoting β-catenin target gene transcription, including genes involved in cell migration and distant metastasis. In *RCC2* knockout cells, β-catenin is ubiquitinated and targeted for proteasomal degradation, reducing its transcriptional activity. Conversely, CD24 influences cytoskeletal organization, impacting cell migration through the regulation of cytoskeleton-associated proteins such as Vimentin. The interplay between RCC2 and CD24 modulates the activation or inhibition of these pathways, contributing to prostate cancer cell migration and metastasis.

Table S1 Human Subject Characteristics

Categories		Total cases
Age y, median (range)		61 (45–75)
PSA (ng/ml), median (range)		7.7 (3.0–49.6)
Tumor stage (TNM)		
T2		20
T3		17
T4 or N+ or M+		41
Gleason score		
G5-6	8	
G7	54	
G8-10	16	

Table S2 Identification of top RCC2-binding partners by mass spectrometry analysis

Name	Ranking	Protein score	Peptides	Size	Molecular mass	Function
sp|P34932|HSP74-HSPA4	13	1301	28	840 amino acids	94331Da	ATP binding; Chaperone-mediated protein complex assembly; Protein import in to mitochondrial outer membrane; Response to unfold protein
sp|P13797|PLST-Plastin-3	28	1045	24	630 amino acids	70811Da	Actin filament binding; Calcium ion binding
sp|P08670|VIME-Vimentin	32	1020	26	466 amino acids	53652Da	Double-stranded RNA binding; Identical protein binding; Keratin filament binding; Protein C-terminus binding; Protein domain specific binding; Scaffold protein binding; Stuctural constituent of cytoskeleton
sp|P68366|TBA4A-Tubulin alpha-4A chain-TUBA4A	70	754	25	448 amino acids	49924Da	GTPase activity; GTP binding; Protein kinase binding; Structural constituent of cytoskeleton
sp|P07237|PDIA1-Protein disulfide isomerase-P4HB	90	686	12	508 amino acids	57116Da	Enzyme binding; Integrin binding; RNA binding; Peptide disulfide oxidoreductase activity
sp|P11586|C1TC-Protein disulfide isomerase-MTHFD1	117	616	12	935 amino acids	101559Da	ATP binding; Formate-tetrahydrofolate ligase activity
sp|Q5VTE0|EF1A3-Putative elongation factor 1-alpha-like 3-Eukaryotic translation elongation factor 1 alpha	137	568	20	462 amino acids	50141Da	GTpase activity; GTP binding; Translation elongation factor activity

**sp|O95757|HS74L- Heat shock 70 kDa protein 4L- HSPA4L**	146	544	12	839 amino acids	94512Da	ATP binding; Protein folding; Response to unfolded protein
**sp|P31040|SDHA- Succinate dehydrogenase [ubiquinone] flavoprotein subunit, mitochondrial**	156	531	13	664 amino acids	72692Da	Electron transfer activity; Flavin adenine dinucleotide binding; Succinate dehydrogenase (ubiquinone) activity
**tr|H0YN42|H0YN42- Annexin (Fragment)- ANXA2**	165	516	8	339 amino acids	38604Da	Calcium-dependent phospholipid binding; Calcium ion binding; Cytoskeletal protein binding; Phospholipase inhibitor activity
**tr|C9J406|C9J406- MICOS complex subunit MIC60-** IMMT	215	447	10	758 amino acids	83678Da	RNA binding; mitochondrial calcium ion homeostasis
**sp|O95678|K2C75- Keratin, type II cytoskeletal 75- KRT75**	218	443	11	551 amino acids	59560Da	Structural molecule activity
**tr|A0A087WVV2|A0A087WVV2-RRBP1- Ribosome-binding protein 1**	244	423	8	1410 amino acids	152456Da	RNA binding; Signaling receptor activity
**tr|F8W6I7|F8W6I7- HNRNPA1- Heterogeneous nuclear ribonucleoprotein A1**	247	409	7	372 amino acids	38747Da	ATP binding; Chaperone-mediated protein complex assembly; Protein import in to mitochondrial outer membrane; Response to unfold protein
**tr|H3BUF6|H3BUF6- Ataxin-2-like protein- ATXN2L**	253	402	8	1075 amino acids	113374Da	Cadherin binding; RNA binding; Regulation of cytoplasmic mRNA processing body assembly; Stress granule assembly

**sp|P05787|K2C8-Keratin, type II cytoskeletal 8 –KRT8**	254	401	10	485 amino acids	53704Da	Protein-containing complex binding; Scaffold protein binding; Strucltural molecule activity
**tr|H0Y8G5|H0Y8G5-Heterogeneous nuclear ribonucleoprotein D0 (Fragment) OS-HNRNPD**	256	398	9	355 amino acids	38434Da	RNA binding; Poly (A) RNA binding
**sp|O43852|CALU-Calumenin**	274	376	9	315 amino acids	37107Da	Calcium ion binding
**tr|A0A1B0GTG2|A0A1B0GTG2- Alpha-aminoadipic semialdehyde dehydrogenase-ALDH7A1**	275	375	7	539 amino acids	58487Da	Oxidoreductase activity; NAD or NADP as aceptor
**tr|Q5H909|Q5H909-Melanoma-associated antigen D2-MAGED2**	285	365	7	606 amino acids	64954Da	Female pregnancy; Platelet degradulation; Renal sodium ion absorption
**tr|A8MUD9|A8MUD9-60S ribosomal protein L7-RPL7**	297	358	7	248 amino acids	29226Da	DNA binding; mRNA binding; Protein homodimerization activity; RNA binding; Structural constituent of ribosome
**sp|Q04695|K1C17-Keratin, type I cytoskeletal 17-KRT17**	299	351	8	432 amino acids	48106Da	MHC class II protein binding; MHC class II receptor activity; Structural contituent of cytoskeleton

Table S3 Specific primary antibodies used in this study

	Antigen	Species	Supplier	Cat #	Dilution
Western blotting (WB)	RCC2	human	abcam	ab70787	3000
	CD24	human	Santa Cruz Biotechnology	sc-70598	1000
	E-cadherin	human	Abcam	ab76055	3000
	Vimentin	human	Cell Signaling Technology	5741	1000
	GTP-RAC1	human	Cytoskeleton	BK035-S	1000
	GTP-ARF6	human	Cytoskeleton	BK033-S	1000
	Ubi	human	Proteintech Rosemont	10201-2-AP	1000
	FLAG	human	Cell Signaling Technology	14793	2000
	GFP	human	Cell Signaling Technology	2956	2000
	(3-catenin	human	Cell Signaling Technology	9562	1000
	(3-catenin (Active)(Ser33/37/Thr41)	human	Cell Signaling Technology	8814	1000
	Phospho-(3-catenin (Thr41/Ser45)	human	Cell Signaling Technology	9565	1000
	Axin2	human	Cell Signaling Technology	2151	1000
	APC	human	Cell Signaling Technology	2504	1000
	Lamin B1	human	Santa Cruz Biotechnology	sc-374015	250
	GAPDH	human	Cell Signaling Technology	5174	5000
	(3-tubulin	human	Santa Cruz Biotechnology	sc-101527	5000
	(3-actin	human	Proteintech	66009-1-Ig	5000
IF	RCC2	human	Cell Signaling Technology	3667	500
	CD24	human	BD Biosciences	555426	200
	Vimentin	human	Cell Signaling Technology	5741	100
	alpha-tubulin	human	Sigma-Aldrich	T6199	500
	Alexa Fluor® 568 phalloidin (for F-actin)	human	Thermo Fisher Scientific	A12380	500
IHC	RCC2	human	Cell Signaling Technology	3667	100
	CD24	human	BD Biosciences	555426	100
	Androgen Receptor	human	Cell Signaling Technology	5153	100
	E-cadherin	human	Cell Signaling Technology	3195	100
	Vimentin	human	Cell Signaling Technology	5741	100
	RCC2	mouse	abcam	ab70788	100
	Androgen Receptor	mouse	Santa Cruz Biotechnology	sc-7305	100
	PTEN	mouse	Cell Signaling Technology	9188	100
	PSA	mouse	Invitrogen	PA5-86200	100
	E-cadherin	mouse	Cell Signaling Technology	3195	100
	Vimentin	mouse	Cell Signaling Technology	5741	100

Table S4 The sequences of primer, sgRNA, and genotyping used in this study

Primer Name	Sequence
**CRISP/Cas9 sgRNAs**	
Human CD24 gRNA1	CACCTTTTATATGAGAGTACATGG
Human CD24 gRNA2	AAACCCATGTACTCTCATATAAAA
Human RCC2 gRNA1	CACCGCCTCCTCATCACCACGGAA
Human RCC2 gRNA2	AAACTTCCGTGGTGATGAGGAGGC
**RT-PCR primers**	
Human RCC2-F	AACAGCAAGCTGCTTACCG
Human RCC2-R	CCCTTCTCATTTCGACCCCA
Human AXIN2-F	GTTGGCTTGTCCAGCAAAACT
Human AXIN2-R	GCTCCTCTTGAAGGACCTGTATC
Human GAPDH-F	CCCCTTCATTGACCTCAACTACAT
Human GAPDH-R	CGCTCCTGGAAGATGGTGA
**CRISP/Cas9 off-target PCR primers**	
MARS-off-target-F	CACTGTGCTCGCTTCCTGGC
MARS-off-target-R	GGAAGGGCGTCCCTTAAGAA
MAPKAPK3-off-target-F	GGAGGTAGACCATCACTGGC
MAPKAPK3-off-target-R	CAGGGATGCCGAGGGGGTGT
CPNE2-off-target-F	AGGGTTTGGGATGAAGGAGG
CPNE2-off-target-R	ATGATGGACATGGGCAGCTT
PRKCD-off-target-F	TCCTTCTGTACGAGATGCTC
PRKCD-off-target-R	TTCTCCAGGATGTCCTTGGA
**Mouse genotyping primers**	
Rcc2-5arm-WTF	GATGCAGGGCCTGAGGTAT
Rcc2-Crit-WTR	CTGCTTAGAGGTCCCATCCA
Rcc2-5mut-R1	GAACTTCGGAATAGGAACTTCG
Pten-oIMR9554	CAAGCACTCTGCGAACTGAG
Pten-oIMR9555	AAGTTTTTGAAGGCAAGATGC
Nkx3-1CreWT-F	CTCCGCTACCCTAAGCATCC
Nkx3-1CreWT-R	GACACTGTCATATTACTTGGACC
Nkx3-1CreMut-F	CAGATGGCGCGGCAACACC
Nkx3-1CreMut-R	GCGCGGTCTGGCAGTAAAAAC
TRAMP-10363	TACAACTGCCAACTGGGATG
TRAMP-10364	CAGGCACTCCTTTCAAGACC
TRAMP-21238	CTGTCCCTGTATGCCTCTGG
TRAMP-21239	AGATGGAGAAAGGACTAGGCTACA
