# Supplementary material for: RCC2 and CD24 cooperate to modulate prostate cancer progression through vimentin ubiquitination and β-catenin activation
Source: J Clin Invest. 2025 Oct 15;135(20):e192883. doi: 10.1172/JCI192883 (PMC12520681; doi:10.1172/JCI192883)

Immunoblot Images

Fig. 2D

IB: GFP

IB: RCC2-Flag (DYK)

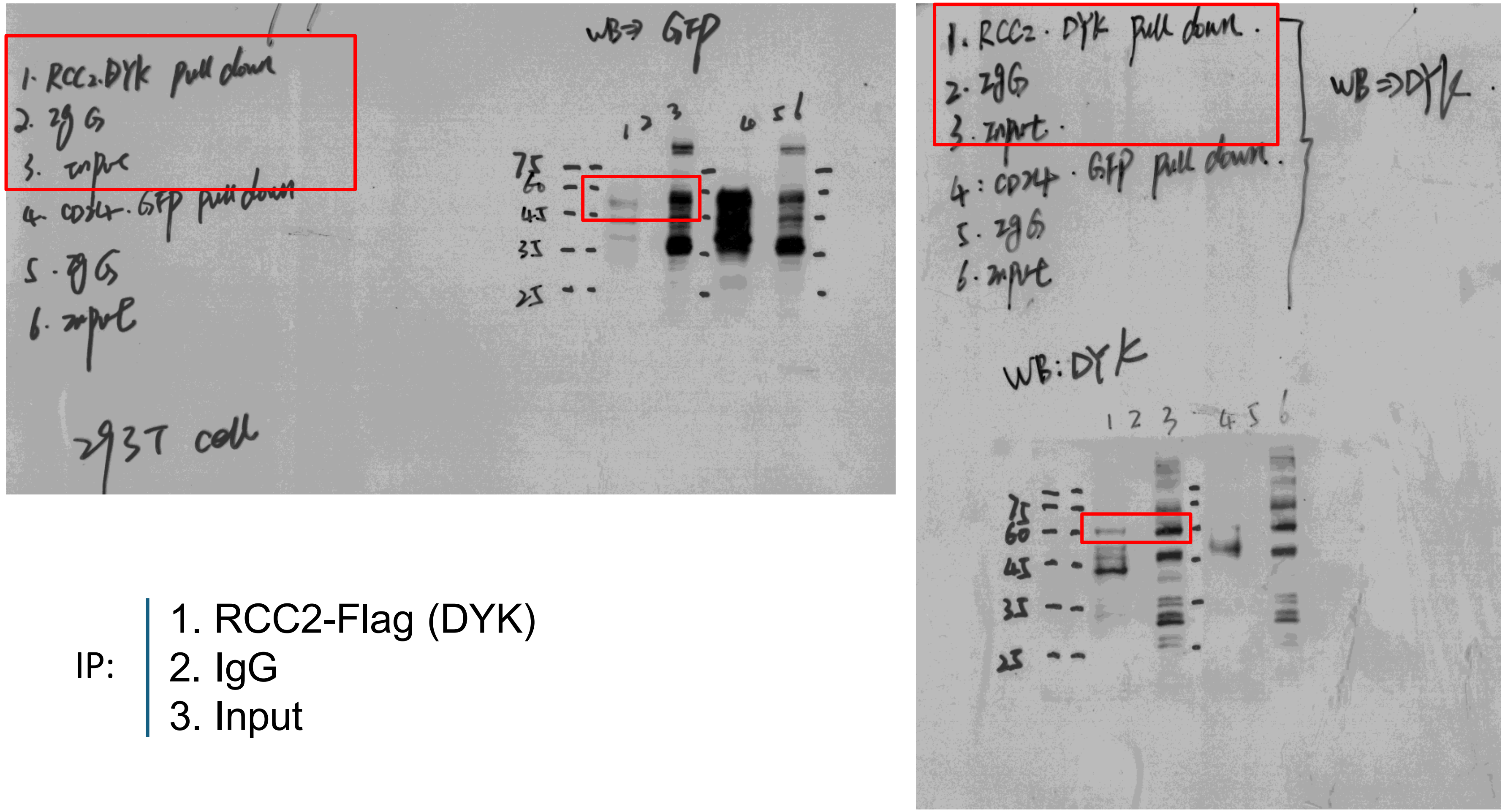

IP:
1. RCC2-Flag (DYK)
2. IgG
3. Input

Fig. 2E

IP:
1. CD24-GFP
2. IgG
3. Input

IB: RCC2-Flag (DYK)

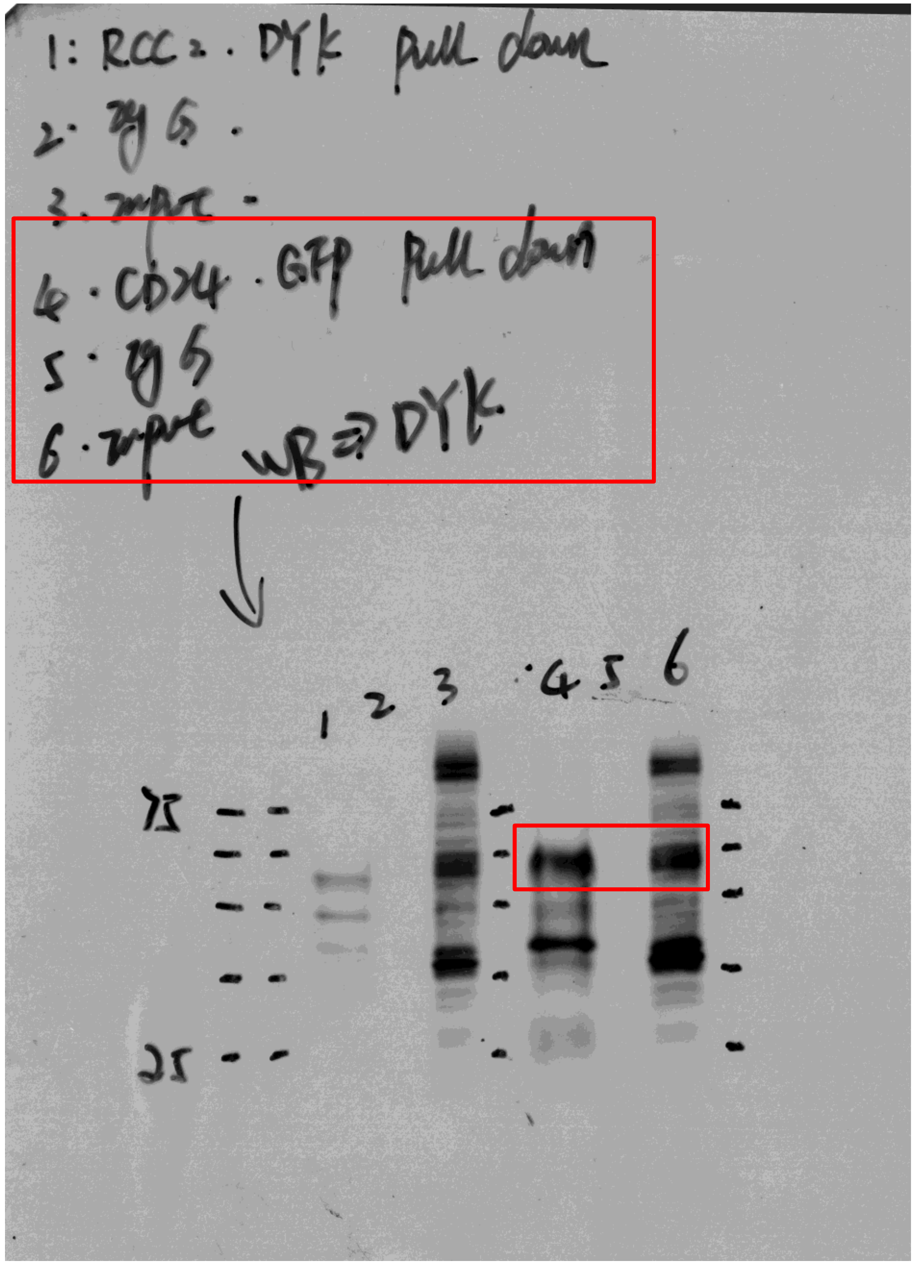

IB: CD24-GFP

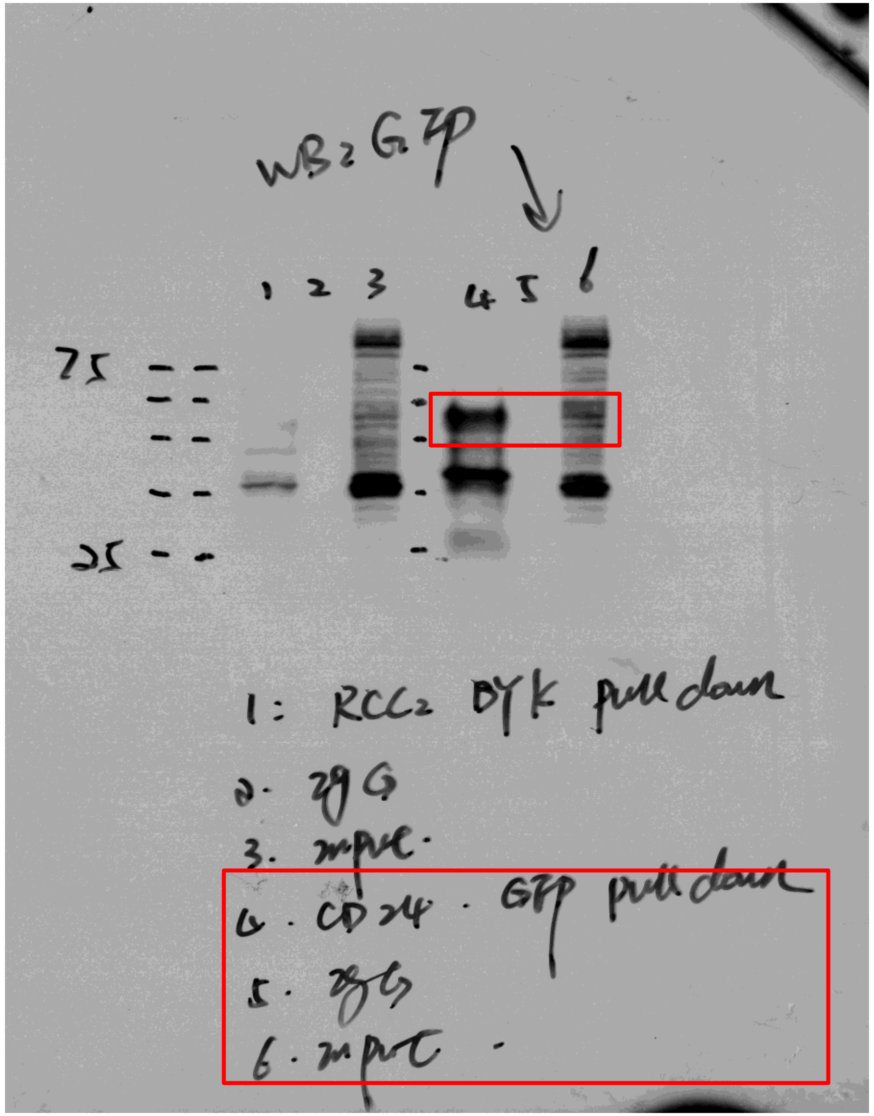

Fig. 2F

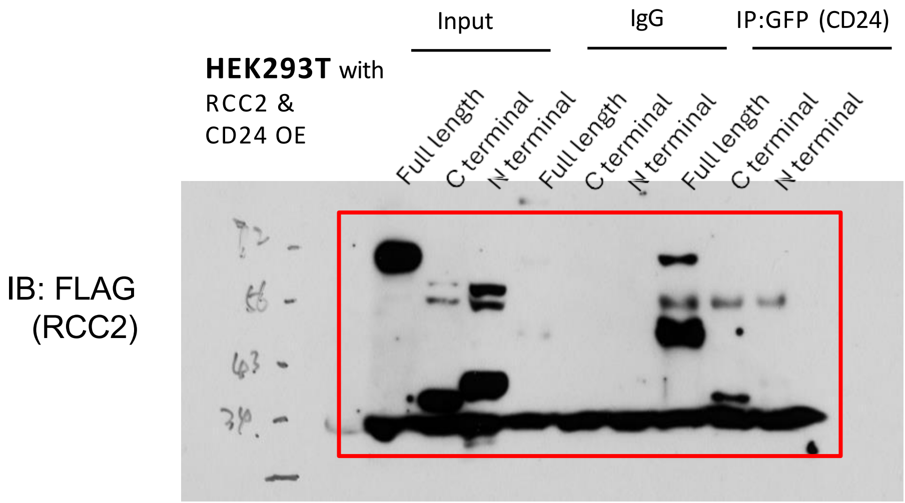
Input
IgG
IP:GFP (CD24)
HEK293T with
RCC2 &
CD24 OE
Full length
C terminal
N terminal
Full length
C terminal
N terminal
Full length
C terminal
N terminal
IB: FLAG
(RCC2)

Fig. 4E

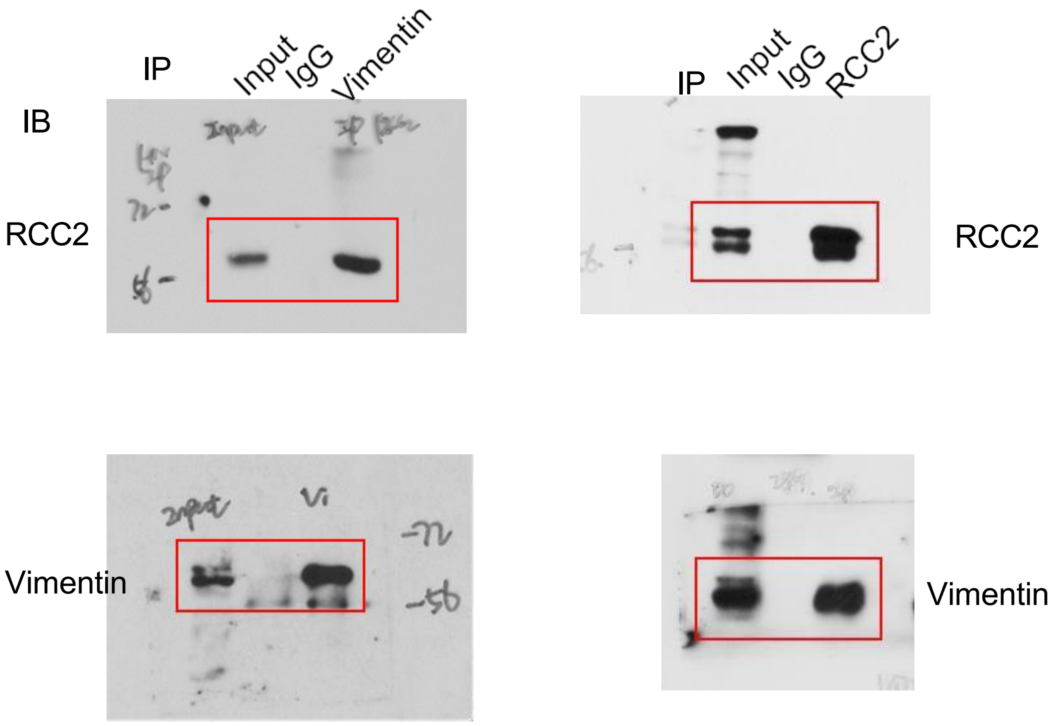
IP
Input
IgG
Vimentin
IB
RCC2
IP
Input
IgG
RCC2
RCC2
Vimentin
Vimentin

Fig. 4F

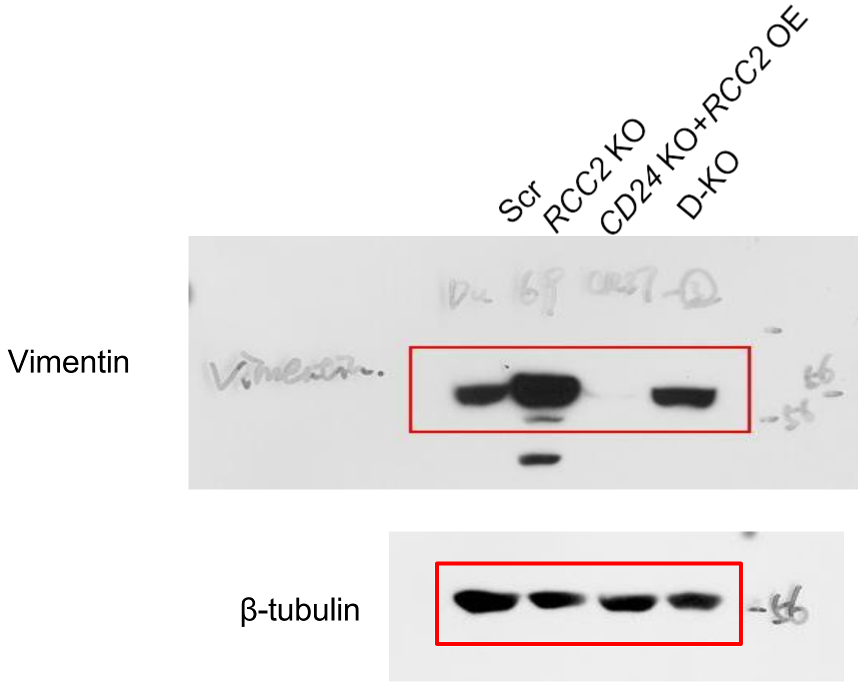
Scr
RCC2 KO
CD24 KO+RCC2 OE
D-KO
Vimentin
β-tubulin

Fig. 4G

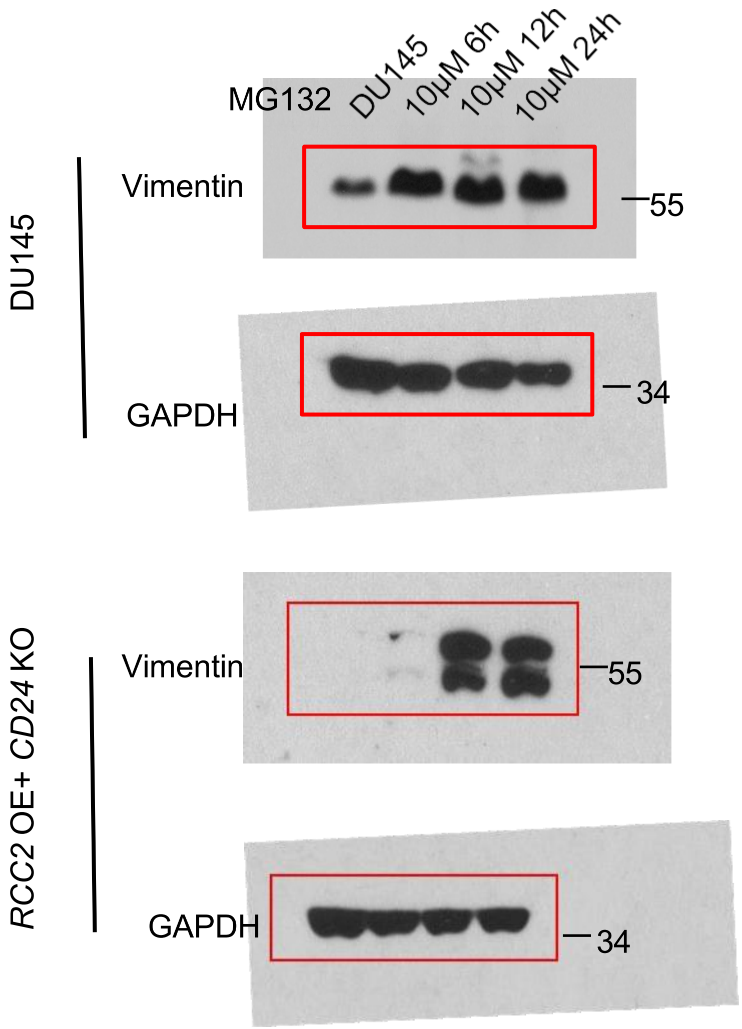

MG132
DU145
10μM 6h
10μM 12h
10μM 24h
DU145
Vimentin
55
GAPDH
34
RCC2 OE+ CD24 KO
Vimentin
55
GAPDH
34

Fig. 4H

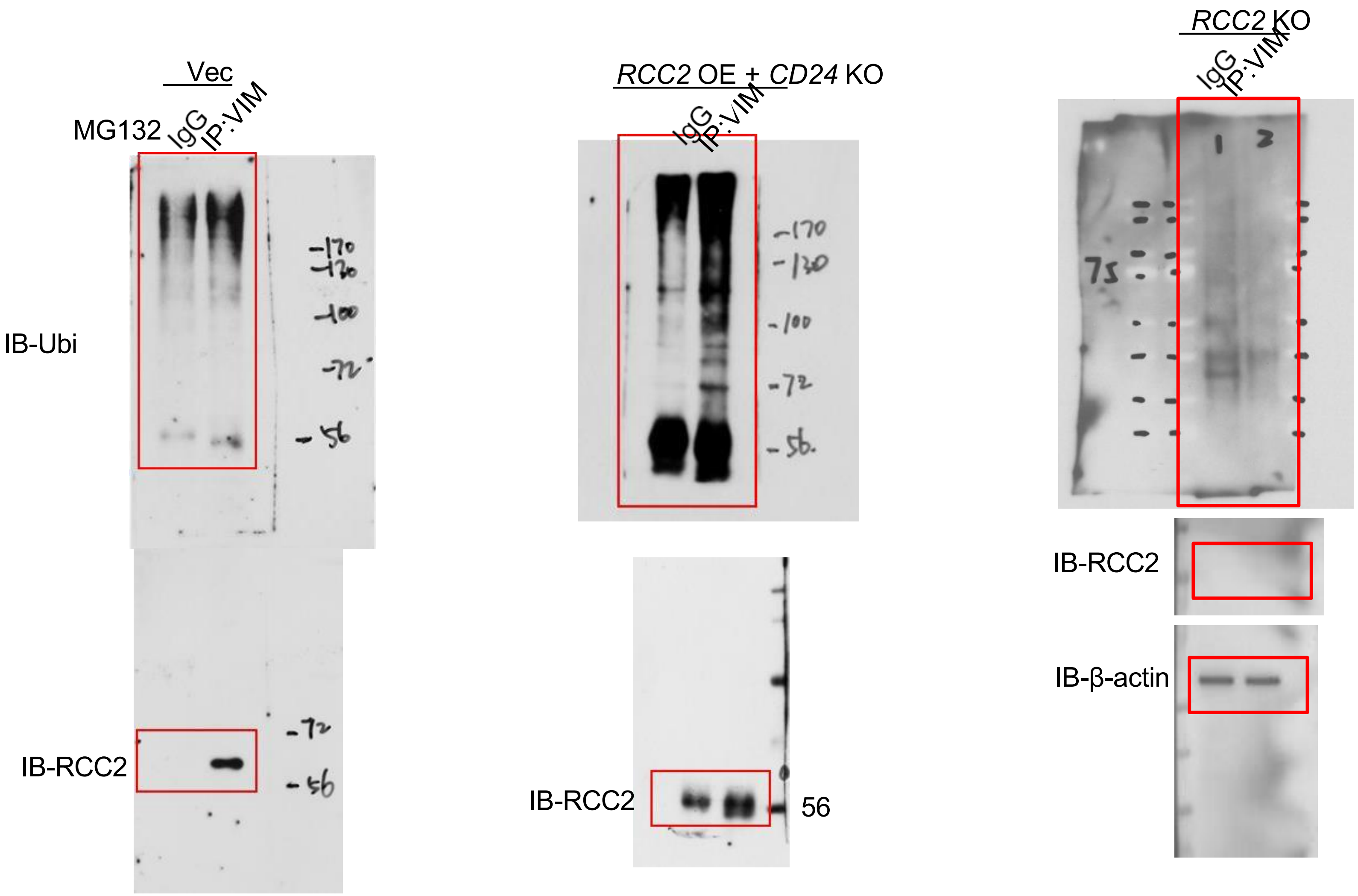
Vec
MG132
IgG
IP:VIM
IB-Ubi
IB-RCC2
RCC2 OE + CD24 KO
IgG
IP:VIM
IB-RCC2
56
RCC2 KO
IgG
IP:VIM
IB-RCC2
IB-β-actin

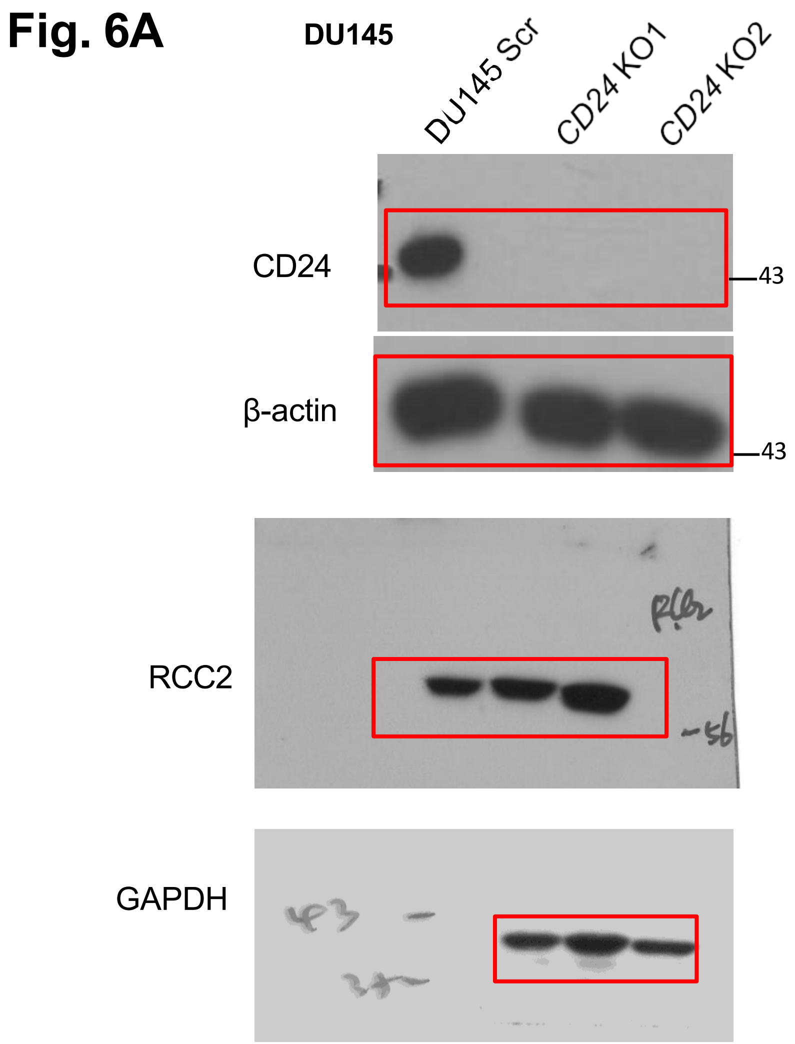
Fig. 6A
DU145
DU145 Scr
CD24 KO1
CD24 KO2
CD24
43
β-actin
43
RCC2
GAPDH

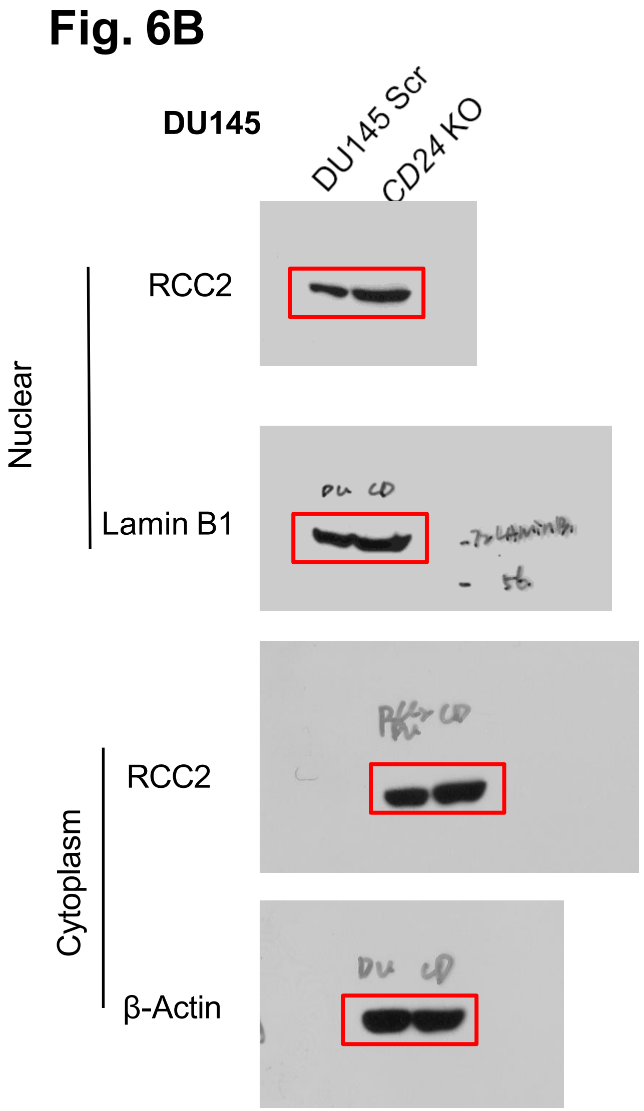
Fig. 6B
DU145
DU145 Scr
CD24 KO
Nuclear
RCC2
Lamin B1
Cytoplasm
RCC2
β-Actin

Fig. 6D Fig. 6E

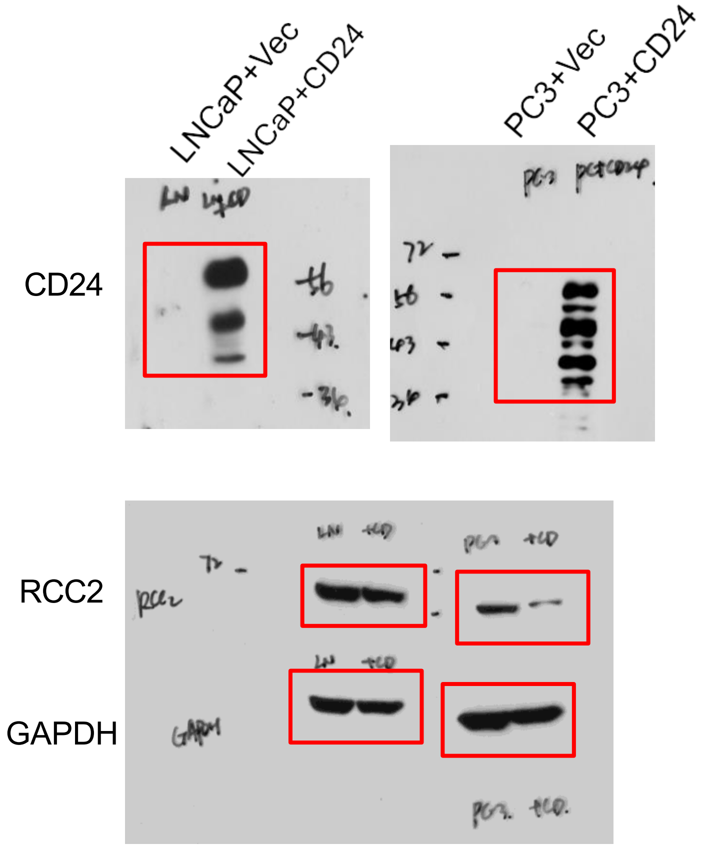

Fig. 6F

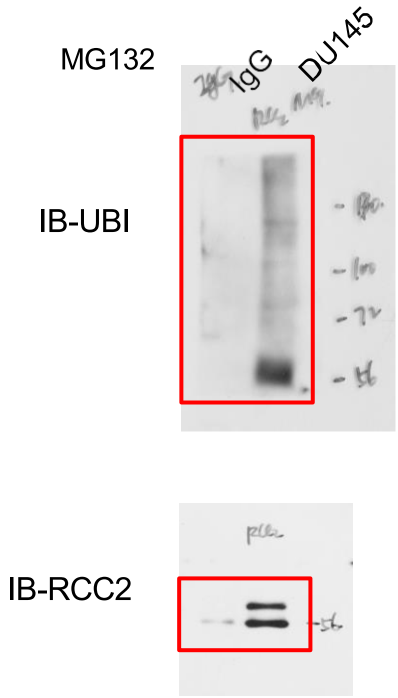

Fig. 6G

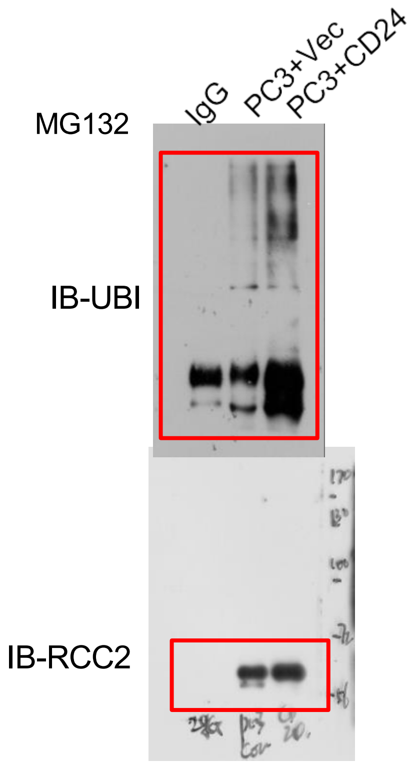

Fig. 7E

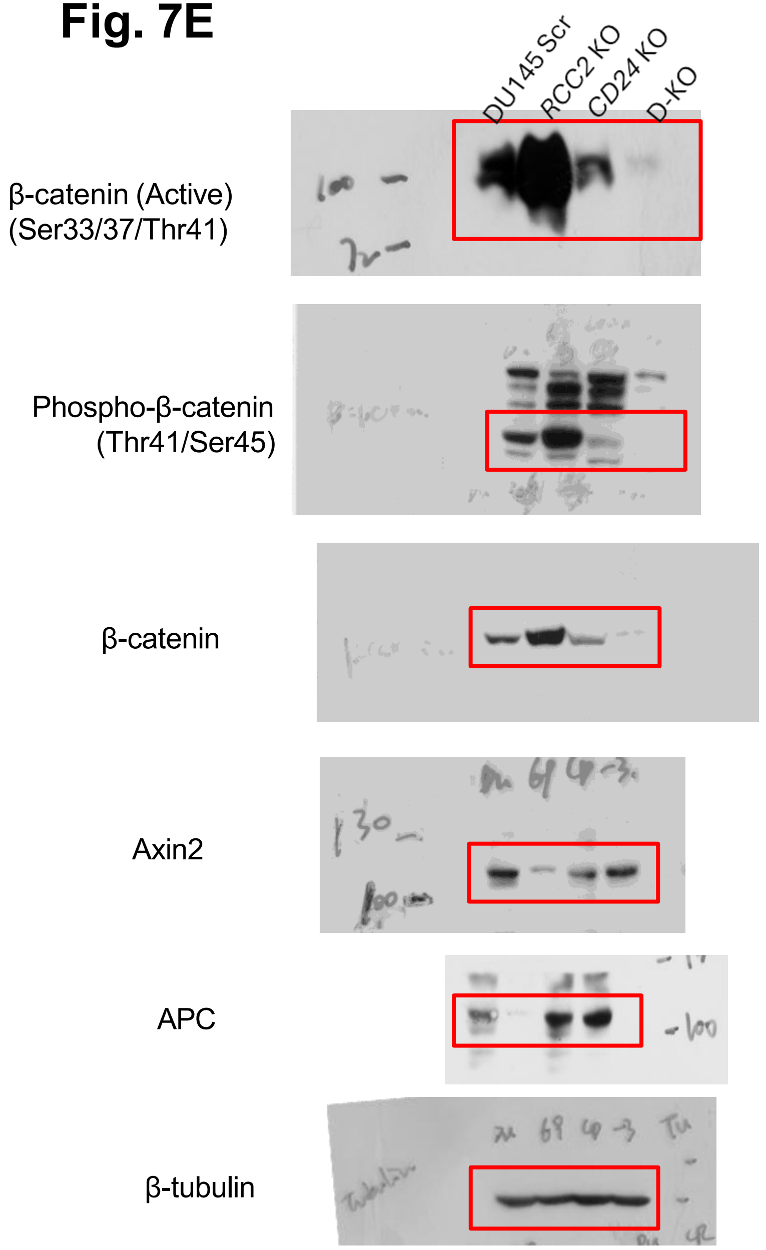
DU145 Scr
RCC2 KO
CD24 KO
D-KO
β-catenin (Active)
(Ser33/37/Thr41)
Phospho-β-catenin
(Thr41/Ser45)
β-catenin
Axin2
APC
β-tubulin

Fig. 7F

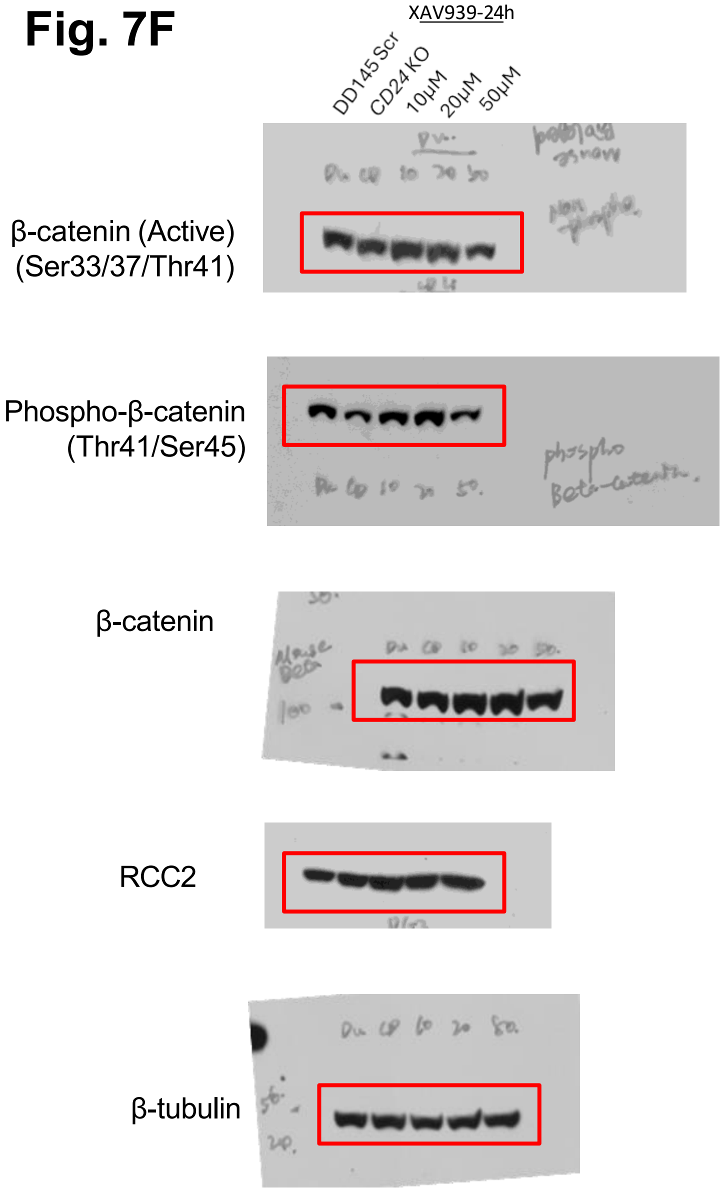
XAV939-24h
DD145Scr
CD24 KO
10μM
20μM
50μM
β-catenin (Active)
(Ser33/37/Thr41)
Phospho-β-catenin
(Thr41/Ser45)
β-catenin
RCC2
β-tubulin

Fig. 7G

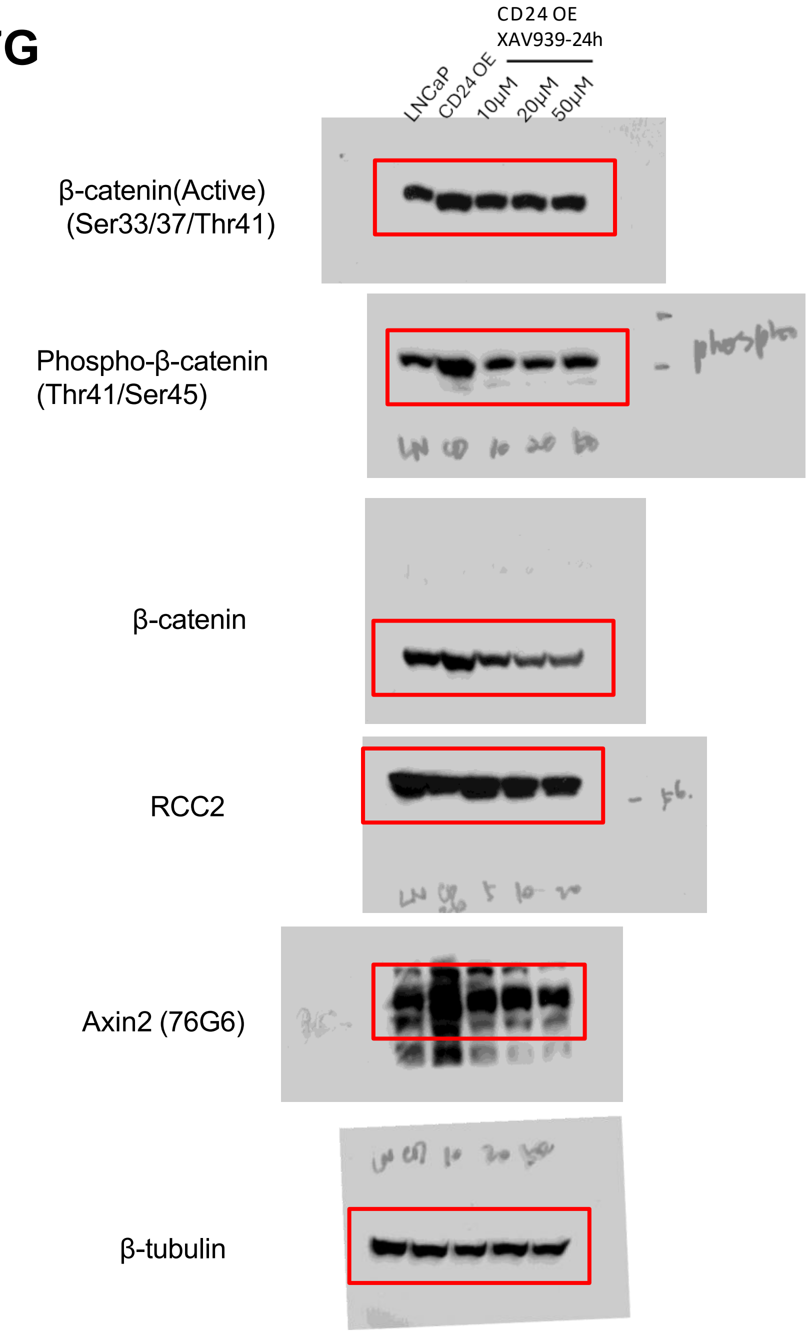
CD24 OE
XAV939-24h
LNCaP
CD24 OE
10μM
20μM
50μM
β-catenin(Active)
(Ser33/37/Thr41)
Phospho-β-catenin
(Thr41/Ser45)
β-catenin
RCC2
Axin2 (76G6)
β-tubulin

Supplemental Fig. 3

Fig. S3A

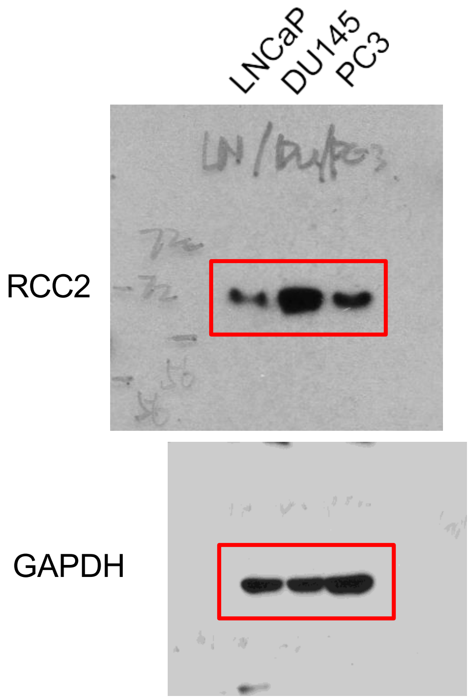

Fig. S3F

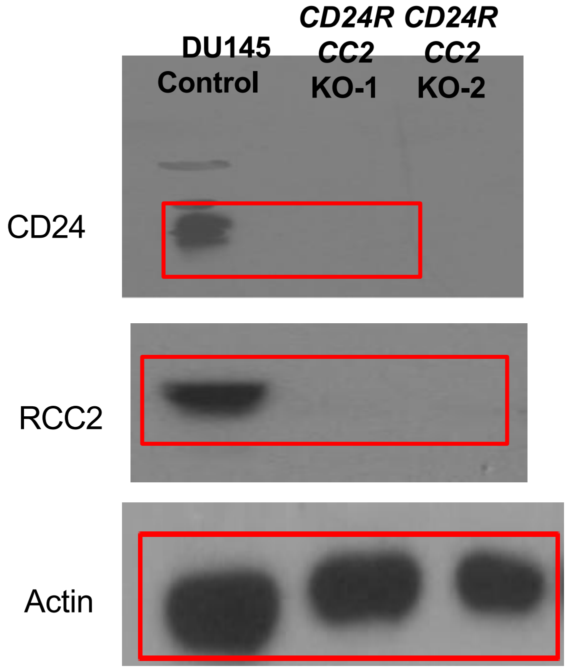

Supplemental Fig. 3

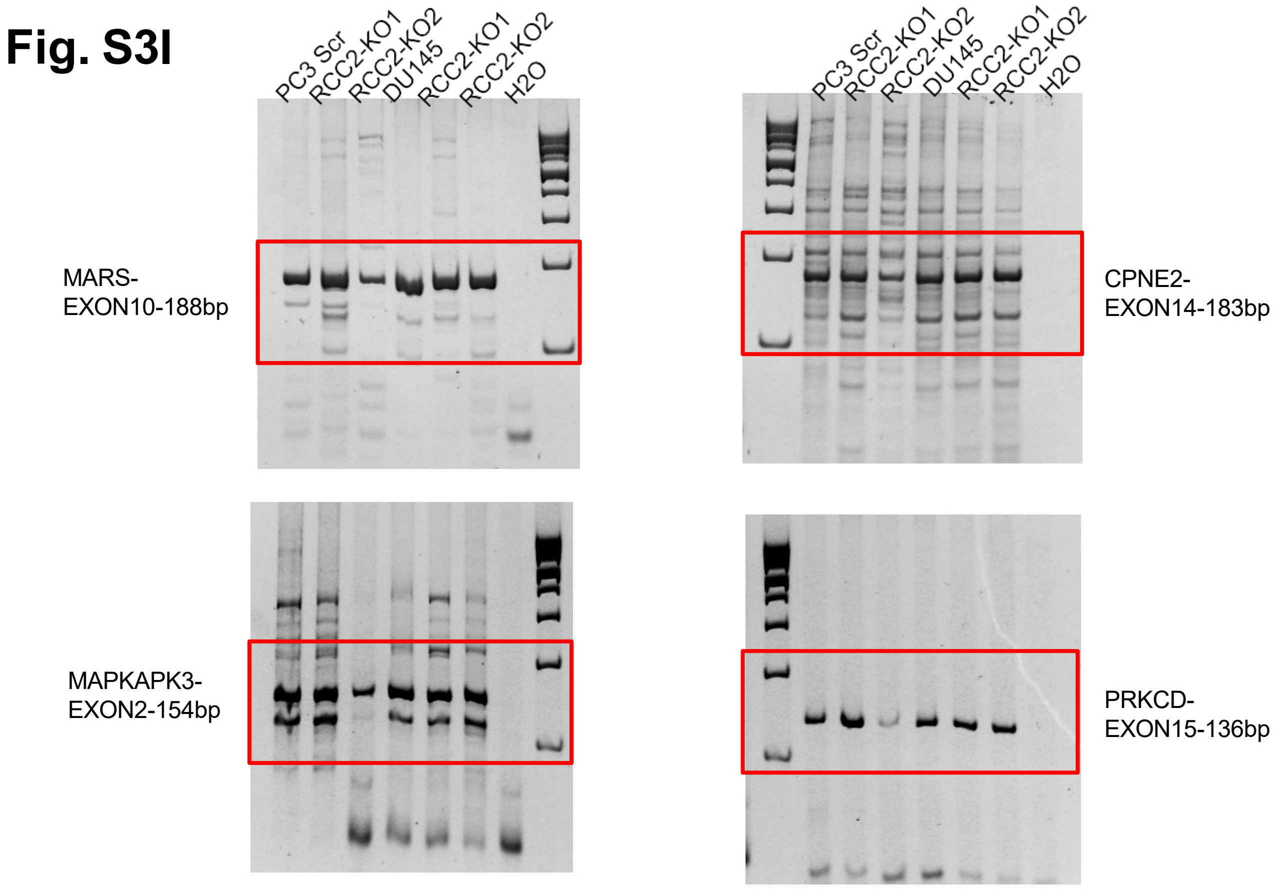

Supplemental Fig. 4

Fig. S4A

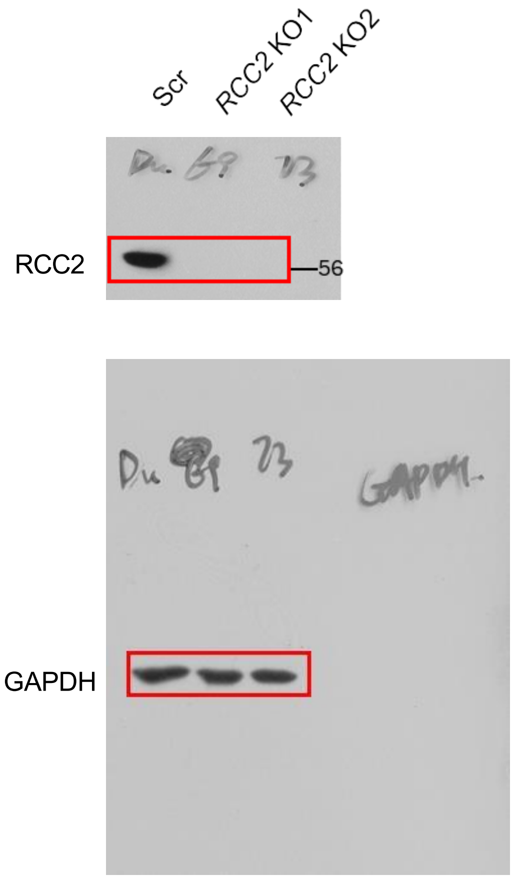

Supplemental Fig. 5A

Fig. S5A

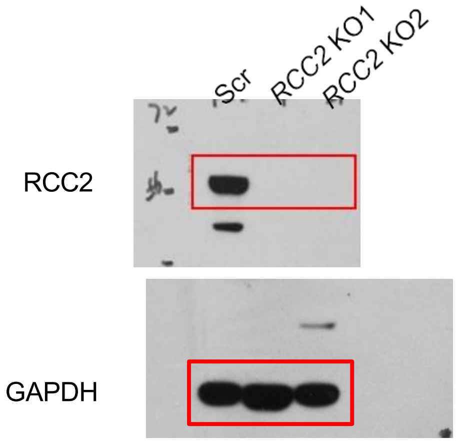

Supplemental Fig. 7

Fig. S7C

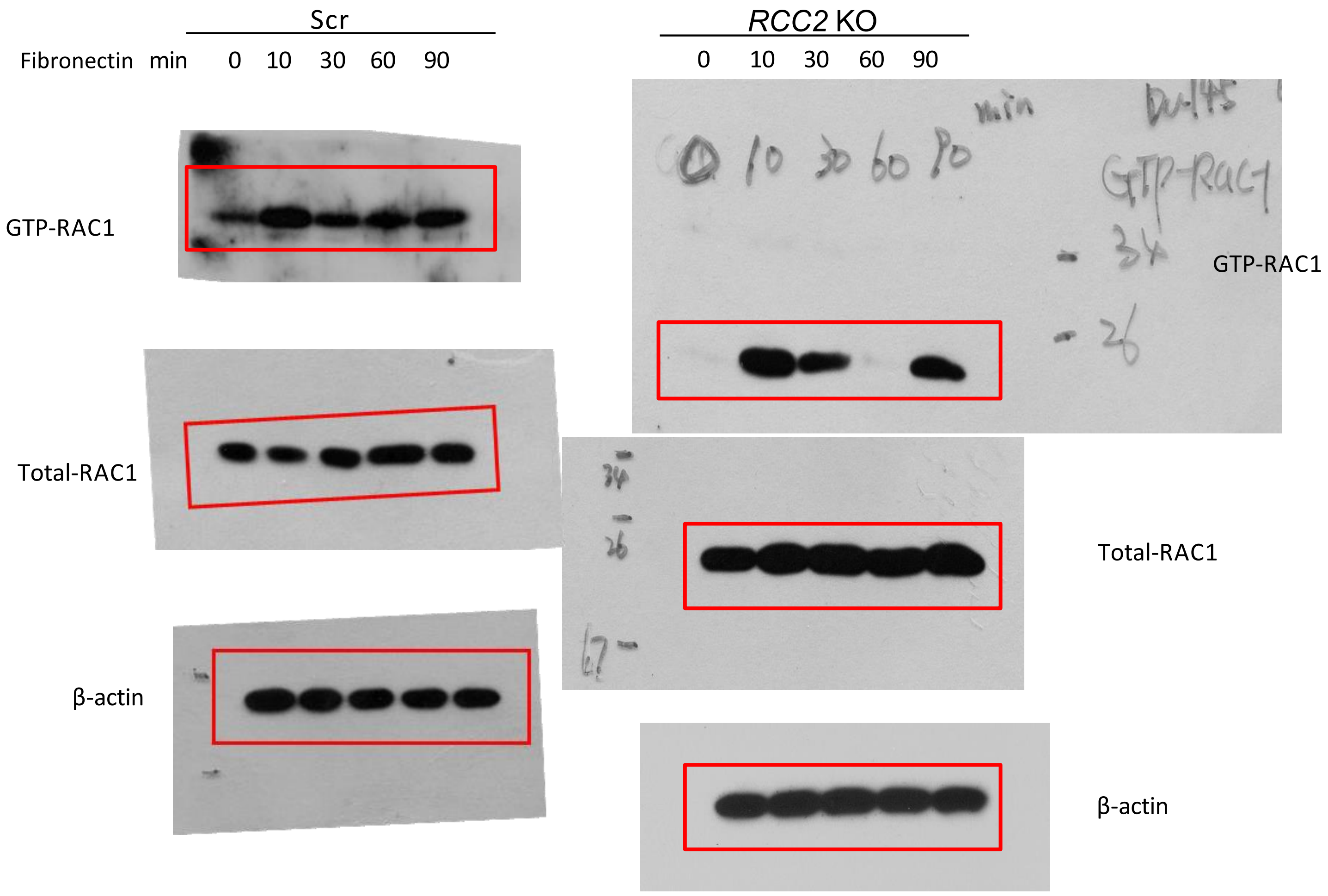

Supplemental Fig. 7

Fig. S7D

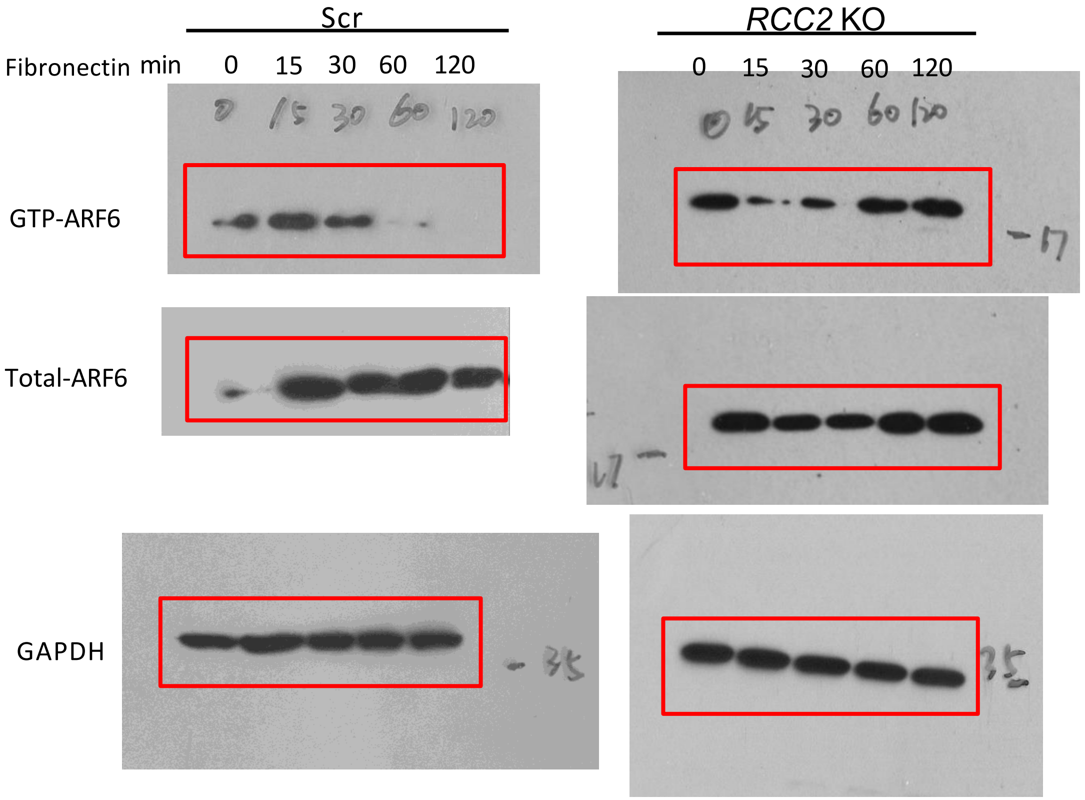

Supplement: Unedited blot and gel images [file jci-135-192883-s279.pdf]
